# Neural control and adaptive neural forward models for insect-like, energy-efficient, and adaptable locomotion of walking machines

**DOI:** 10.3389/fncir.2013.00012

**Published:** 2013-02-13

**Authors:** Poramate Manoonpong, Ulrich Parlitz, Florentin Wörgötter

**Affiliations:** ^1^Bernstein Center for Computational Neuroscience, The Third Institute of Physics, Georg-August-Universität GöttingenGöttingen, Germany; ^2^Max Planck Research Group Biomedical Physics, Max Planck Institute for Dynamics and Self-OrganizationGöttingen, Germany; ^3^Institute for Nonlinear Dynamics, Georg-August-Universität GöttingenGöttingen, Germany

**Keywords:** efference copy, central pattern generators, sensory feedback, recurrent neural networks, local leg control, walking gait, autonomous robots

## Abstract

Living creatures, like walking animals, have found fascinating solutions for the problem of locomotion control. Their movements show the impression of elegance including versatile, energy-efficient, and adaptable locomotion. During the last few decades, roboticists have tried to imitate such natural properties with artificial legged locomotion systems by using different approaches including machine learning algorithms, classical engineering control techniques, and biologically-inspired control mechanisms. However, their levels of performance are still far from the natural ones. By contrast, animal locomotion mechanisms seem to largely depend not only on central mechanisms (central pattern generators, CPGs) and sensory feedback (afferent-based control) but also on internal forward models (efference copies). They are used to a different degree in different animals. Generally, CPGs organize basic rhythmic motions which are shaped by sensory feedback while internal models are used for sensory prediction and state estimations. According to this concept, we present here adaptive neural locomotion control consisting of a CPG mechanism with neuromodulation and local leg control mechanisms based on sensory feedback and adaptive neural forward models with efference copies. This neural closed-loop controller enables a walking machine to perform a multitude of different walking patterns including insect-like leg movements and gaits as well as energy-efficient locomotion. In addition, the forward models allow the machine to autonomously adapt its locomotion to deal with a change of terrain, losing of ground contact during stance phase, stepping on or hitting an obstacle during swing phase, leg damage, and even to promote cockroach-like climbing behavior. Thus, the results presented here show that the employed embodied neural closed-loop system can be a powerful way for developing robust and adaptable machines.

## 1. Introduction

Walking animals, like locusts, stick insects, and cockroaches, can traverse diverse terrains in an energy-efficient way. During traversing, their locomotion can also adapt to deal with terrain changes. Furthermore, their movements are elegant and versatile. These capabilities are the result of the coupling of biomechanics (Dickinson et al., 2000) and neural control. For instance, the appropriate biomechanical structures of body and legs of a cockroach (Ritzmann et al., 2004) allows it to walk naturally, deal with minor disturbances during traversing rough terrain, and even climb over relatively high obstacles as compared to its size. While biomechanics allows for such capabilities, neural control, on the other hand, combines information from different sensor modalities and provides coordinated outputs to many motor joints (Büschges, 2005; Grillner, 2006; Cruse et al., 2009; Mulloney and Smarandache, 2010; Fuchs et al., 2011). This process is fast and adaptive which leads to the generation of locomotion and adaptation.

During the last few decades, roboticists have tried to imitate such natural properties with artificial legged locomotion systems. Several of them have paid attention on the biomechanical design of such systems to have animal-like properties (Cham et al., 2002; Iida and Pfeifer, 2004; Lewinger et al., 2005; Kingsley et al., 2006; Schneider et al., 2012). Others have focused on sensorimotor coordination and control for locomotion and adaptation by using different approaches including machine learning algorithms (Lee et al., 2006; Erden and Leblebicioglu, 2008), classical engineering control techniques (Brooks, 1986; Shkolnik and Tedrake, 2007), and biologically inspired control mechanisms (Beer et al., 1997; Kuo, 2002; Lewis and Bekey, 2002; Dürr et al., 2003; Ekeberg et al., 2004; Cruse et al., 2007; Kimura et al., 2007; Spenneberg and Kirchner, 2007; Amrollah and Henaff, 2010; Daun-Gruhn and Büschges, 2011; Harischandra et al., 2011; Lewinger and Quinn, 2011; von Twickel et al., 2012). With increasing machine complexity, integrating more behaviors, and obtaining adaptability, the control problems become more challenging.

Artificial neural networks (ANNs) appear appropriate for such control problems due to their intrinsically distributed architecture, their capability to integrate new behaviors, as well as synaptic learning (Beer et al., 1997; Dürr et al., 2003; Ekeberg et al., 2004; Cruse et al., 2007; Kimura et al., 2007; Amrollah and Henaff, 2010; Daun-Gruhn and Büschges, 2011; Lewinger and Quinn, 2011; Harischandra et al., 2011; von Twickel et al., 2012). In addition they have a number of excellent properties as follows. They are able to build a controller as a composition of different neural modules to produce desired motor behaviors (von Twickel et al., 2012). And, they are conceptually close to biological systems compared to other solutions. In particular recurrent neural networks (RNNs) exhibit dynamical behavior (oscillatory, hysteresis, chaotic patterns, etc.) for generating basic rhythmic locomotion behavior (Beer et al., 1997; Kimura et al., 2007; Amrollah and Henaff, 2010; Daun-Gruhn and Büschges, 2011; von Twickel et al., 2012). Considering this, here we exploit the features of ANNs to develop locomotion control for walking machines. This is based on a modular structure consisting of different neural modules having main functions that follow three key mechanisms found in animal locomotion (Holst and Mittelstaedt, 1950; Meyrand et al., 1991; Cruse et al., 1998; Katz, 1998; Bläsing and Cruse, 2004; Cruse et al., 2009; Harris-Warrick et al., 2011): (1) central mechanisms [i.e., central pattern generators (CPGs)] for generating basic rhythmic motions, (2) sensory feedback (i.e., afferent-based control) for shaping the motions, and (3) internal forward models (i.e., efferent-based control) for sensory prediction and walking state estimations. While these three key mechanisms are essential for locomotion control as found in biological legged systems, only individual instances of them had been successfully applied to artificial ones (Beer et al., 1997; Ishiguro et al., 2003; Cruse et al., 2007; Kimura et al., 2007; Spenneberg and Kirchner, 2007; Amrollah and Henaff, 2010; Schroeder-Schetelig et al., 2010; Harischandra et al., 2011; Lewinger and Quinn, 2011; Owaki et al., 2012; von Twickel et al., 2012), thereby providing partial solutions. A few studies have applied all these mechanisms to animal-like legged robots to achieve complex behavior and adaptability (Lewis and Bekey, 2002). However, the mechanisms have been often used for active two-legged walking (Lewis and Simo, 2001).

Taking all these mechanisms into account for the design of our adaptive neural locomotion control leads to robust walking behavior in many situations. Furthermore, the controller can generate a multitude of walking patterns (e.g., 20 patterns), insect-like leg movements, and energy-efficient and adaptable locomotion for a biomechanical six-legged walking machine, like the AMOS II^1^ robot used here. It also allows AMOS II to cope with leg damage and even promote cockroach-like climbing behavior. Besides the complex behavior generation, the rationales behind this study are also: (1) to give a better understanding of how a CPG mechanism with neuromodulation, sensory feedback, and adaptive internal forward models with efference copies can be combined in artificial legged locomotion systems and (2) to emphasize that the generated behaviors require the coupling of biomechanics (i.e., physical structure) and neural mechanisms with sensory feedback embedded in an embodied neural-closed loop system. The work presented here extends our previous works (Manoonpong et al., 2007, 2008b; Steingrube et al., 2010) by modifying a chaotic CPG (Steingrube et al., 2010) into a CPG with neuromodulaiton leading to more gaits and smoother and faster switching between them compared with the chaotic CPG. It also introduces for the first time local leg feedback and adaptive forward models as well as their combination with the CPG in robust walking behaviors.

The following section describes the technical specification of the six-legged walking machine AMOS II used for the experiments, followed by adaptive neural locomotion control. The controller is developed to generate versatile and adaptable locomotion of walking machines. The experimental results are shown in section 3. Discussion is given in section 4.

## 2. Materials and methods

All the experiments of this work were carried out with the physical six-legged walking machine AMOS II. Thus, the first section describes its biomechanical setup, followed by details of the adaptive neural locomotion controller and its components which are the main contribution of this work. Here, some results are described alongside the introduced components from which they mainly derive because this provides a better understanding of their functionalities.

### 2.1. The walking machine platform AMOS II (biomechanics)

**Figure 1:**
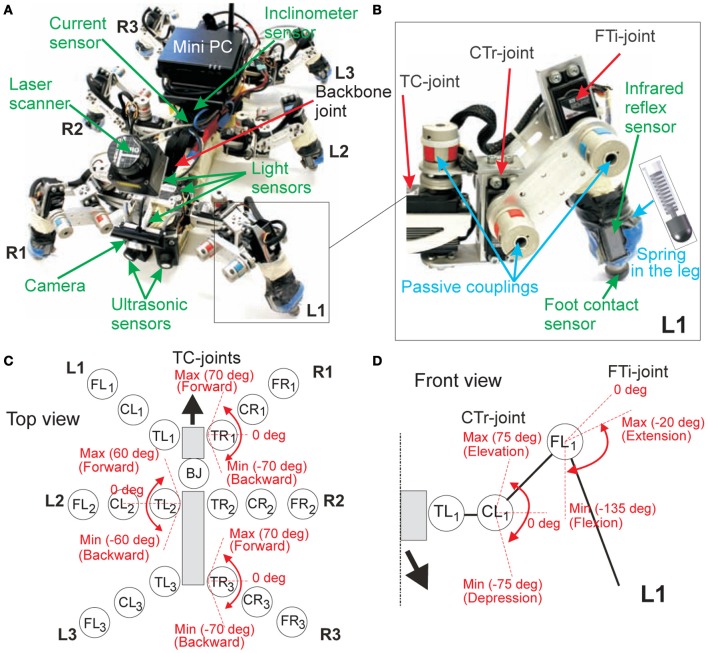
**The biologically-inspired six-legged walking machine AMOS II. (A)** AMOS II with its sensors. **(B)** Examples of components at the left front leg (L1). **(C)** The location of all motor joints on AMOS II and the maximum and minimum angles of the TC-joints of the right front (R1), left middle (L2), and right hind (R3) legs (top view). The remaining legs on the opposite side have the same ranges; i.e., the range of L1 = R1, the range of R2 = L2, and the range of L3 = R3. **(D)** The maximum and minimum angles of the CTr- and FTi-joints of L1 (front view). The remaining legs perform the same joint angle ranges. Abbreviations are: TR1, CR1, FR1 = TC-, CTr-, and FTi-joints of the right front leg (R1); TR2, CR2, FR2 = right middle leg (R2); TR3, CR3, FR3 = right hind leg (R3); TL1, CL1, FL1 = left front leg (L1); TL2, CL2, FL2 = left middle leg (L2); TL3, CL3, FL3 = left hind leg (L3); BJ = a backbone joint.

In order to explore and test the performance of the proposed adaptive neural locomotion control in a physical system, the six-legged walking machine AMOS II is employed (Figure 1A). It is an improved version of our previous six-legged walking machine AMOS (Steingrube et al., 2010).

AMOS II has six identical legs. Each leg has three joints (Figure 1B): the thoraco-coxal (TC-) joint enables forward (+) and backward (−) movements, the coxa-trochanteral (CTr-) joint enables elevation (+) and depression (−) of the leg, and the femur-tibia (FTi-) joint enables extension (+) and flexion (−) of the tibia (Figures 1C,D). The morphology of these multi-jointed legs is modeled on the basis of a cockroach leg (Zill et al., 2004) but the tarsus segments are ignored. Each tibia contains a spring compliant element to substitute part of the function of the tarsus; i.e., absorbing the impact force during touchdown on the ground. In addition, a passive coupling is installed at each joint (Figure 1B) in order to yield passive compliance and to protect the motor shaft. The maximum and minimum ranges of the joint movements of the legs are shown in Figures 1C,D. In a normal walking condition (e.g., walking on flat terrain), we set the default joint movements so that its body is very close to the ground (i.e., low center of mass) and its body falls to the ground before taking the next step during normal walking. However, for walking over rough terrains, these ranges will be automatically shifted such that AMOS II lifts its body up for better locomotion. This walking strategy is inspired by insect walking, like that of a cockroach (Alexander, 1982; Ritzmann et al., 2004, 2012) and it also ensures stability when confronting leg damage.

**Figure A1:**
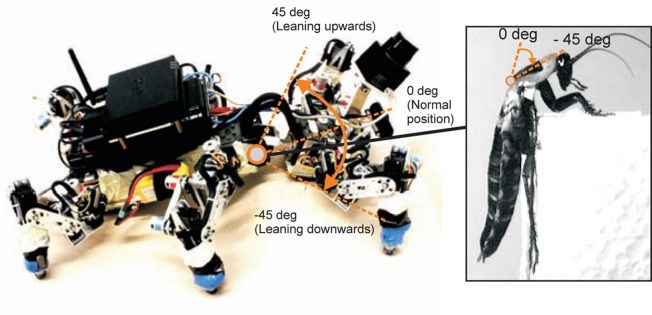
**The six-legged walking machine AMOS II inspired by the morphology of a cockroach. Left:** Climbing position of AMOS II with a body flexion joint. **Right:** Climbing position of a cockroach. It can bend its front body downwards to keep the legs close to the surface of an object for an optimum climbing position and even to prevent unstable actions (modified from Ritzmann et al., 2004).

The body of AMOS II consists of two segments: a front segment where two front legs are installed and a central body segment where the two middle and the two hind legs are attached. They are connected by one active backbone joint (BJ) inspired by the invertebrate morphology of the American cockroach's trunk (Figure A1). This BJ can rotate around the lateral or transverse axis in a range between −45° (minimum downward position) and +45° (maximum upward position). It stays at zero degree during walking and it leans upwards and bends downwards while climbing. In total, AMOS II has 19 active joints (three at each leg, one BJ). They are driven by digital servomotors (HSR-5990 TG) delivering a stall torque of 2.9 Nm at 5 V. In addition, the body joint torque is tripled by using a gear to achieve a more powerful body joint motion. Besides the motors, AMOS II has 21 sensors: two ultrasonic sensors (US) at the front body part, six foot contact (FC) sensors in its legs, six infrared reflex (IR) sensors at the front of its legs, one current sensor (CS) and one inclinometer (IM) sensor inside the body, and three light dependent (LD) sensors, one USB camera (CM) and one laser scanner (LS) on the front body part (Figure 1). These sensors are used to generate stimulus induced behavior (like, photo tropism and obstacle avoidance) as well as versatile, energy-efficient, and adaptable locomotion. The USB camera is used for terrain classification and the LS is used to measure obstacle height in order to distinguish between a wall and a surmountable obstacle.

We use a Multi-Servo IO Board (MBoard) installed inside the body to digitize all sensory input signals except the CM and LS signals. We also use it to generate a pulse-width-modulated signal to control the position of the servomotor. For experiments here, the MBoard is connected to a personal computer (PC) where the CM and LS are directly connected and a neural locomotion controller is implemented. The communication between a PC and the MBoard is accomplished via an RS232 interface at 57.6 kb/s. Electrical power supply for all servomotors, the MBoard, and all sensors is given by lithium polymer batteries with a voltage regulator producing a stable 5 V supply.

### 2.2. Adaptive neural locomotion control

**Figure 2:**
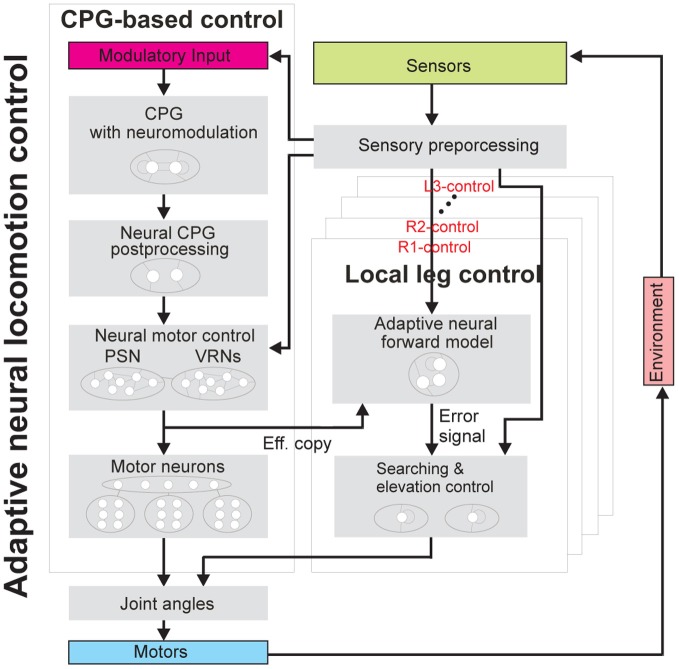
**Adaptive neural locomotion control.** The controller generates insect-like, energy-efficient, and adaptable locomotion of AMOS II. This adaptive neural closed-loop controller consists of one CPG-based control unit and six local leg control units (R1-, R2-, R3-, L1-, L2-, and L3-control) (see text for functional description and Figure A2 for the complete circuit). Abbreviations are referred to Figure 1.

The adaptive neural locomotion control (Figure 2) has been developed based on a modular structure. It consists of two main components: CPG-based control and local leg control. The CPG-based control basically coordinates all leg joints of AMOS II, thereby generating insect-like leg movements and a multitude of different behavioral patterns. The patterns include forward/backward walking, turning left and right, and insect-like gaits. These gaits allow for energy-efficient locomotion on different terrains. All these patterns can be autonomously controlled by exteroceptive sensors, like a camera, a LS, and US. While the CPG-based control provides versatile autonomous behaviors, the local leg control using proprioceptive sensory feedback (like FC sensors) adapts the movement of an individual leg of AMOS II to deal with a change of terrain, losing of ground contact during stance phase, or stepping on or hitting an obstacle during swing phase.

Here, the CPG-based control of the entire system has four components: (1) a CPG mechanism with neuromodulation for generating different periodic signals, (2) neural CPG postprocessing for shaping the CPG signals to obtain smooth leg movements, (3) neural motor control consisting of two additional different networks [phase switching network (PSN) and velocity regulating networks (VRNs)] for controlling walking direction (forward/backward and turning), and (4) motor neurons with delay lines for sending final motor commands to all leg joints of AMOS II.

For the local leg control, it has only two components for each leg: (1) an adaptive neural forward model transforming the motor signal (efference copy) generated by the CPG into an expected sensory signal for estimating the walking state and (2) elevation and searching control for adapting leg motion (e.g., extension/flexion and elevation/depression).

**Figure A2:**
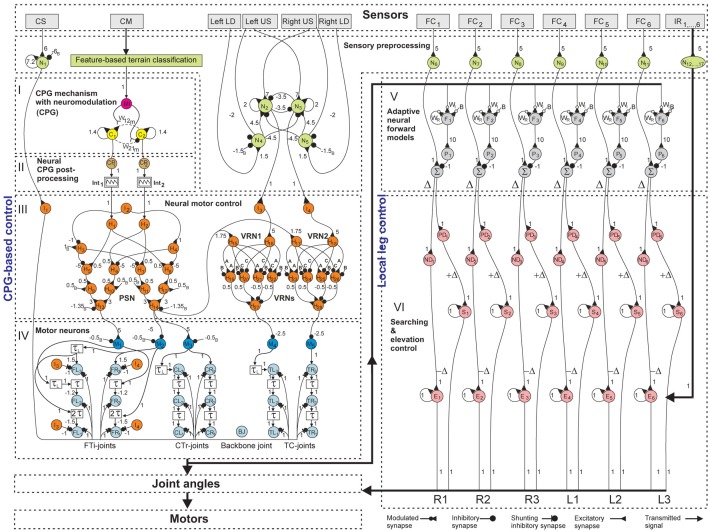
**The complete circuit of adaptive neural locomotion control**.

All neurons of the control network (Figures 2, A2) are modeled as discrete-time non-spiking neurons. They are updated with a frequency of approximately 27 Hz. The activity *a*_*i*_ of each neuron develops according to:

(1) $$a_{i}(t)=\sum_{j=1}^{n}W_{ij}o_{j}(t-1)+B_{i},\;i=1,\ldots ,n,$$

where *n* denotes the number of units, *B*_*i*_ an internal bias term or a stationary input to neuron *i*, *W*_*ij*_ the synaptic strength of the connection from neuron *j* to neuron *i*. The output *o*_*i*_ of all neurons of the network is calculated by using the hyperbolic tangent (tanh) transfer function, i.e., *o*_*i*_ = tanh(*a*_*i*_), ∈ [−1, 1], except for the CPG postprocessing neurons using a step function, the motor neurons using piecewise linear transfer functions, and neurons in searching and elevation control using a linear transfer function.

### 2.3. CPG-based control

The structure of this control unit is based on our previous sensor-driven CPG-based controller (Steingrube et al., 2010) in which a chaotic CPG is used as a main component. While the chaotic CPG can produce different periodic output signals including a chaotic one, only a few number of gaits (e.g., five different gaits) and a chaotic motion have been realized for hexapod locomotion (Steingrube et al., 2010). Furthermore, switching between these gaits cannot be immediately achieved but requires a few steps and the transition is non-smooth. This is because the system has to switch to a chaotic state first before obtaining a new periodic pattern.

**Figure 3:**
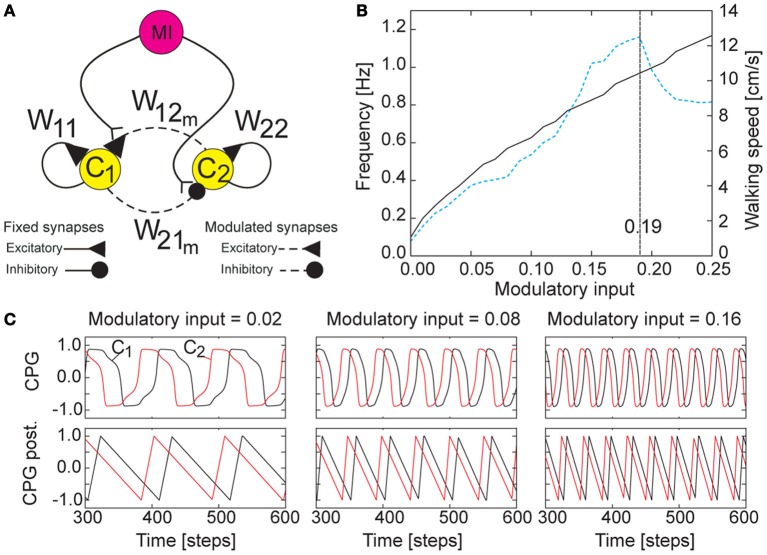
**CPG mechanism with neuromodulation. (A)** Wiring diagram of the CPG circuit. The extrinsic modulatory input *MI* alters the synaptic weights of the CPG, thereby modulating the CPG outputs. The synaptic weights are set as *W*_11,22_ = 1.4, *W*_12_*m*__ = 0.18 + *MI*, *W*_21_*m*__ = −0.18 − *MI*. **(B)** The resulting eigenfrequency of the outputs of the CPG (black solid line, left scale) and the walking speed of AMOS II (blue dashed line, right scale) with respect to *MI*. Here *MI* is increased by 0.01. If *MI* is smaller than 0.0 the network dynamics exhibits only fixed point attractors; i.e., oscillations are switched off. Recall that the CPG network is updated with a frequency of approximately 27 Hz (i.e., one time step is ≈0.037 s). **(C)** Examples of the asymmetrical periodic outputs of the CPG (top) where *MI* is set to 0.02, 0.08, and 0.16. The signals differ in phase by π/2 and are shaped by neural CPG postprocessing such that smooth ascending and descending signals are obtained for motor control (bottom). This kind of asymmetrical periodic signals is appropriate for walking found in insects where swing (ascending slope) and stance (descending slope) phases differ in duration, being intrinsically asymmetry (Wilson, 1966).

Thus to overcome this drawback, in this study we modify the chaotic CPG to a simpler CPG mechanism with neuromodulation. It is inspired by biological findings (Meyrand et al., 1991; Katz, 1998; Harris-Warrick et al., 2011) (see the section 4 for more details). It provides a large number of periodic output patterns including a chaotic one, resulting in a large number of walking patterns (i.e., more than five stable gaits). It also allows fast and smooth switching between patterns. The circuit consists of two neurons *i* ∈ {1, 2}, fully connected (Figure 3A). The discrete-time dynamics of the activity states *a*_*i*_ and the output states *o*_*i*_ of the circuit follows Equation (1) and a tanh transfer function, respectively. Their initial states are set to a small positive value, e.g., 0.1. An extrinsic modulatory input *MI* is introduced and projected to the synaptic connections of the neurons (Figure 3A), thereby modulating the outputs of the CPG (Figures 3B,C). *MI* will be controlled by a sensory signal (see the section 3). According to this, the synaptic weights are described as:

(2) $$W_{11,22}=W_{d0},$$

(3) $$W_{12_{m}}=W_{d1}+MI,$$

(4) $$W_{21_{m}}=-(W_{d1}+MI),$$

where *W*_11,22_ are fixed synapses and *W*_12_*m*_,21_*m*__ are modulated synapses. *W*_*d*0_ and *W*_*d*1_ are the default synaptic weights, which are used to create basic periodic signals. They need to be selected in accordance with the dynamics of the system that generates periodic or quasi-periodic attractors (Pasemann et al., 2003).

**Figure 4:**
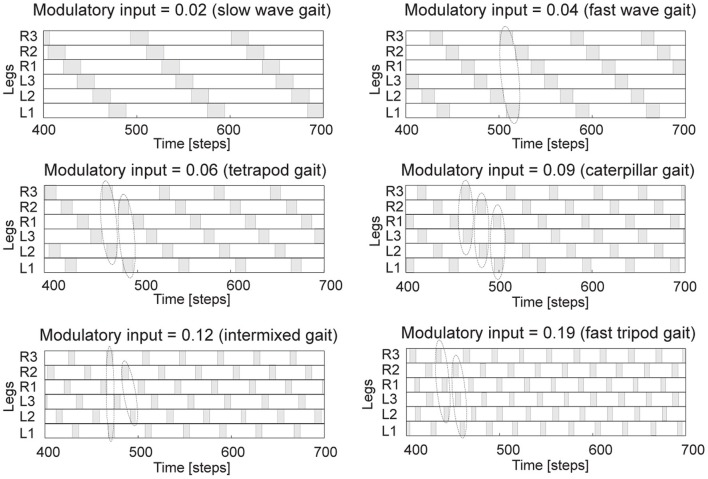
**Examples of six different gaits generated by the CPG.** They are observed from the motor signals of the CTr-joints (Figure 1). White areas indicate ground contact or stance phase and gray areas refer to no ground contact during swing phase. As frequency increases, some legs step in pairs (dashed enclosures). We encourage readers to see also Figure 3 and **Video S2** for, e.g., 20 walking patterns with respect to *MI* = 0.0, 0.01, …, 0.19. Note that one time step is ≈ 0.037 s.

We empirically adjust and set the parameters to *W*_*d*0_ = 1.4 and *W*_*d*1_ = 0.18. This parameter setup with *MI* = 0.0 results in a very low frequency of the periodic outputs. Increasing *MI* will increase the frequency of the outputs (see black solid line in Figure 3B). The investigation of AMOS II walking on a flat floor using this CPG shows that its walking speed is proportional to the value of *MI*; i.e., increasing *MI* leads to the increasing of walking speed (see blue dashed line in Figure 3B). However, the walking speed will decrease if *MI* is grater than 0.19. This is because the output frequency is too high such that the motors of AMOS II cannot follow the driving frequency properly^2^. Interestingly, together with neural motor control and a delay line mechanism embedded in the motor neuron module (described below), AMOS II shows different walking patterns at the different values of *MI* (e.g., 20 patterns) where some of these patterns show similar gaits but differ in stepping frequency in the swing and stance phases. Figure 4 shows examples of six different patterns or gaits: slow wave gait (*MI* = 0.02), fast wave gait (*MI* = 0.04), tetrapod gait (*MI* = 0.06), caterpillar gait (*MI* = 0.09), intermixed gait (*MI* = 0.12), and fast tripod gait (*MI* = 0.19). Some of them are similar to insect gaits (Wilson, 1966) and allow for energy-efficient locomotion on particular terrains (see the section 3). Here we use visual information to trigger the most energy-efficient gait while AMOS II traverses different terrains. Visual information is obtained from a terrain classification system consisting of the USB camera of AMOS II (Figure 1A) and an online feature-based terrain classification algorithm. The camera acquires terrain images while the classification algorithm (i.e., image processing) extracts local features of the images using Scale Invariant Feature Transform (SIFT) (Lowe, 2004), encodes the features using the Bag of Words (BoW) technique (Zhang et al., 2010), and then classifies the words using Support Vector Machines (SVMs) with a radial basis function kernel (Cortes and Vapnik, 1995). The output of the algorithm provides terrain information used to set *MI* of the CPG, thereby triggering the corresponding pre-mapped energy-efficient gait (see the section 3).

Fast and smooth switching between gaits in a comparison to our previous chaotic CPG can be seen at **Video S1**. In principle, for the AMOS II system, a transition state from one stable gait to another stable gait using the CPG with neuromodulation requires about 2 s while it needs about 5 s when using the chaotic CPG. This fast switching between gaits is required for situations like escaping from an attack or danger (i.e., fast changing from a slow wave gait to a fast tripod gait). Note that the change of the modulation value occurs instantaneously where the CPG with neuromodulation immediately switches from one frequency to a new frequency. However, the system requires a longer time for a new gait to emerge because of delay lines (described below) transmitting the CPG signals to the motor neurons.

The outputs of the CPG are passed to motor neurons through two hierarchical subcomponents or modules: neural CPG postprocessing and neural motor control. The neural CPG postprocessing (Figure 2), which directly receives the CPG outputs, consists of postprocessing neurons with a threshold value of 0.85 and integrator units (Figure A2). Specifically, the neurons are for signal shaping while the integrator units are for obtaining continuous signals with asymmetry of ascending and descending slopes (Figure 3C). At first the CPG outputs get transformed by the neurons which produce the step function outputs with high (+1) or low (−1) value. Time intervals of the high and low outputs are counted. The high and low outputs are converted to continuous signals with ascending and descending slopes, respectively. The conversion is done by dividing the integrated high and low outputs by the time intervals part. Since the counting of the time intervals is subsequent, each slope is calculated using the time intervals of the previous period. Finally, the integrator outputs are scaled to the range between −1.0 and 1.0. For different frequencies of the CPG, the time intervals are different, thereby generating different ascending and descending slopes (Figure 3C).

Note that the CPG with the neural CPG postprocessing presented here has certain advantages over a classical solution (e.g., constructing CPG signals directly by hand or using a simple wave-generator). This is because the CPG, derived from a RNN with two neurons, in principle exhibits various dynamical behaviors (e.g., periodic patterns, chaotic patterns, and hysteresis effects) which can be exploited for locomotion control (Manoonpong et al., 2008a; Steingrube et al., 2010). While the network can generate various output patterns, the neural CPG postprocessing is used to only translate these output signals into smooth continuous signals (e.g., saw-tooth signals) for motor control and does not change the network dynamics. In fact, the CPG and its postprocessing are independent; therefore, one could also apply different postprocessing mechanisms to shape or transform the CPG outputs into other periodic forms if required. In this neural approach, we can simply change the gaits (flexibility) and obtain various patterns including chaotic motions^3^ (versatility) by only changing the network parameters (i.e., synaptic weights and bias terms). Furthermore, one could also apply learning mechanisms (with an additional neuron) to the CPG such that the CPG can be entrained by sensory feedback in order to adapt to the feedback pattern and memorize it (Nachstedt et al., 2012). This will lead to the adaptivity of the gaits. Implementing this adaptivity on the AMOS II system is one of our major plans for future work. All these features (flexibility, versatility, and adaptivity) would be difficult to be achieved by a classical solution.

**Figure 5:**
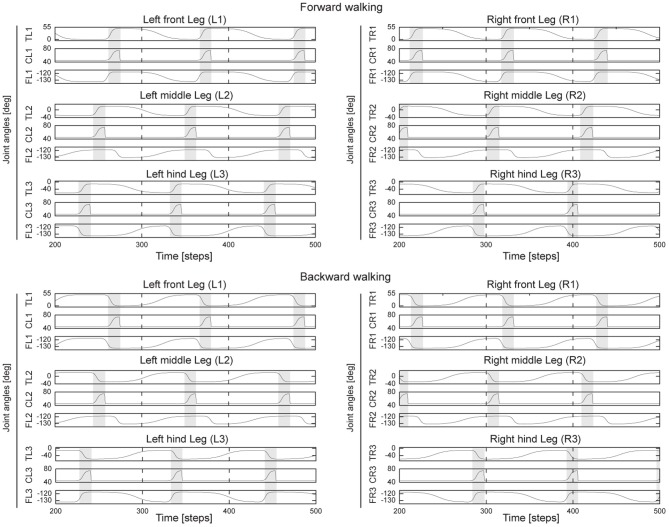
**Angles of the TC-, CTr-, and FTi-joints of all legs during forward and backward walking.** For turning right, all left legs show similar pattern as forward walking while all right legs show similar patterns as backward walking, and vice versa for turning left. All joint angles are in degrees (Figures 1C,D). Gray and white areas indicate the swing and stance phases, respectively. Here *MI* of the CPG is set to 0.02, thereby generating low frequency periodic signals (Figure 3C) and resulting in a slow wave gait (Figure 4). For this gait, the legs swing one by one from hind to front. Note that due to the non-linear neurons of the PSN and VRNs, they further shape the postprocessed CPG signals (Figure 3C) such that the legs decelerate at the beginning of stance phase to avoid large impact force and afterwards they slightly accelerate to produce the propelling force (see, e.g., the TC joint movements). Abbreviations are referred to Figure 1. One time step is ≈ 0.037 s.

The neural motor control, which receives the postprocessed CPG outputs, consists of two different neural networks: one PSN and two VRNs. All neuron outputs of these networks are given by a hyperbolic tangent (tanh) transfer function. The PSN is a generic feedforward network (see Figure A2 for the network structure). This network is designed by hand and consists of 4 hierarchical layers with 12 neurons. The synaptic weights and bias terms of the network are determined in a way that they do not change the periodic form of input signals (i.e., the postprocessed CPG outputs) and keep the amplitude of the signals as high as possible. Thus, all synaptic weights and bias terms were set to 0.5, which will convert the signals in the linear domain of the transfer function, except the synaptic weights and bias terms of the output neurons. They were set to 3.0 and −1.35, respectively, in order to amplify the signals and to shift the offset of the final output signals such that they have their center at zero. The complete network and parameters (i.e., all synaptic weights and bias terms) are shown in Figure A2. As a result, the network can switch the phase of the CPG outputs to lead or lag behind each other by π/2 in phase with respect to a given input for walking sideways [see Steingrube et al. (2010) and Manoonpong et al. (2008b) for more details]. It also provides additional fine tuning of the phase of the CPG outputs to achieve a proper phase shift between the CTr- and FTi-joints leading to insect-like leg movements (Figure 5).

The two VRNs are also simple feed-forward networks (see Figure A2 for the network structure). The network is derived from a multiplication of two values in the range *x*, *y* ∈ [−1, 1]. It was constructed by four hidden neurons, which are connected with an output neuron. The network was trained by using the backpropagation algorithm (Rumelhart et al., 1986). The resulting network parameters (synaptic weights and bias terms) are shown in Figure A2. It approximately works as a multiplication operator. Each VRN controls the three ipsilateral TC-joints on one side. Since the VRNs function qualitatively like a multiplication function (Manoonpong et al., 2007), they have capability to increase or decrease the amplitude of the TC-joint signals and even reverse them with respect to their control inputs. Controlling the TC-joint signals in this way results in various walking directions, like forward/backward, turning left/right, turning in different radians, or curve walking in forward and backward directions [see Manoonpong et al. (2008b) for walking experiments].

Using exteroceptive sensors, like US (Figure 1), together with a neural sensory preprocessing network (see the network *N*_2,3_ in Figure A2) where the network processes the US and provides a final resulting turning signal to the VRNs, allows AMOS II to autonomously avoid obstacles and to escape from a corner and even a deadlock situation (**Video S3**). Currently the network (Figure A2) has fixed synaptic weights resulting in a hard-wired anticipatory behavior with a fixed turning angle in front of the obstacles for avoiding them. Instead one could also apply a learning mechanism [e.g., Hebbian learning and synaptic scaling (Tetzlaff et al., 2011)] to adapt the synaptic weights of the network. This would enable AMOS II to learn to anticipate an obstacle and perform different turning behaviors depending on environmental complexity.

Note that the PSN and VRNs have been developed using a neural approach since this allows for adaptation and the use of standard (neural) learning (e.g., backpropagation) to modify the networks' properties and it is also close to biological systems. For example, there is strong evidence for a phase shifting property found in inter-segmental neurons in the connective elements of a cockroach (Pearson and Iles, 1973). Phase relationships between these neurons can change as would be required for emulating the functionality of our PSN. Studies by Akay et al. (2007) show that in stick insect locomotion motorneuron pools are able to not only drive protractor (swing) and retractor (stance) muscle activities but also reverse their activities leading to the change of locomotion directions (e.g., from walking forward to backward and vice versa). The functionality of these motorneuron pools is directly reproduced by our VRN which controls and reverses motor signals. In addition, another specific functionality of the VRN, namely that of regulating the magnitude of the motor signals allowing for different moving speeds, has been already found in another study (Gabriel and Büschges, 2007). This study suggests that in stick insects there are neurons that receive synaptic input, which modifies their activity according to the walking speed of the animal. This input seems specific to only these neurons and it arises via local pre-motor inter-neurons, which could, thus, represent the VRN interneurons as suggested by our network. In addition to this, the PSN and VRNs are generic and transferable. As suggested by their names, the PSN and VRN serve a general purpose (e.g., “phase switching”) largely regardless of the robot's specific embodiment. Due to modularity, the PSN and VRN are typically independent of each other in their functioning and do not influence or become influenced by other components. Thus, they can be combined to form controllers of different types of robots (Manoonpong et al., 2007, 2008b; Steingrube et al., 2010; Chadil et al., 2011) where they do not require fine tuning for the specific system in which they are employed.

Finally, the outputs of the PSN and VRNs are sent to the motor neurons through delay lines (Figure A2). The ipsilateral lag is determined by a delay τ (i.e., 16 time steps or ≈0.6 s) and the phase shift between both left and right sides is given by a delay τ_*L*_ (i.e., 48 time steps or ≈2 s). These delays are independent of the CPG signals. This setup leads to biologically motivated leg coordination since the legs on each side perform phase shifted waves of the same frequency (Wilson, 1966). The frequency of the waves is defined by *MI* of the CPG. The connections to the motor neurons are similar to our previous work (Steingrube et al., 2010) except the ones to the FTi-motor neurons. They are modified here (Figure A2) to be more similar to insect-like leg movements (Ekeberg et al., 2004; Cruse et al., 2009). Figure 5 illustrates all leg movements during forward and backward walking. During forward walking, in the swing phase the FTi-joints of the front and middle legs extend while the ones of the hind legs flex. In the stance phase, the FTi-joints of the front legs gradually flex to pull the body forward while the ones of the hind legs gradually extend to also push it forward. For the middle legs, the FTi-joints combine both actions of the FTi-joints of the front and hind legs. They flex rapidly and early during the stance phase in order to pull the body since in this period the legs are at an anterior position [i.e., positive TC-joint angles (Figure 1C)]. Afterwards, they stay flexed and then gradually extend in order to push the body since in this period the legs are at a posterior position [i.e., negative TC-joint angles (Figure 1C)]. These biologically-inspired leg movements (Ekeberg et al., 2004; Cruse et al., 2009) provide more propelling force, resulting in an increased walking speed of AMOS II by ≈15% compared with the fixed FTi-joint version (Steingrube et al., 2010). These movements are reversed for backward walking. We encourage readers to also see the video showing the leg movements of AMOS II at **Video S4**. Since the generated leg movements are independent of other influences, similar movements exist in all gaits. It is important to note that the leg movements shown here, however, are still not completely similar to insect leg movements. This can be further improved by applying additional components, i.e., muscle models (Xiong et al., 2012), to obtain a smoother foot path and to come closer to insect-like leg movements.

### 2.4. Local leg control

While the CPG-based control in principle can generate a multitude of different behavioral patterns and insect-like locomotion (i.e., leg movements and gaits) without sensory feedback, it cannot adapt an individual leg to deal with a change of terrain, losing of ground contact during stance phase, or stepping on or hitting an obstacle during swing phase. This adaptable locomotion is necessary for traversing rough terrain or climbing over obstacles. To address this issue, we introduce here local leg control consisting of two components: (1) an adaptive neural forward model and (2) elevation and searching control. These two components are applied to each leg of AMOS II (see Figures 2, A2).

**Figure 6:**
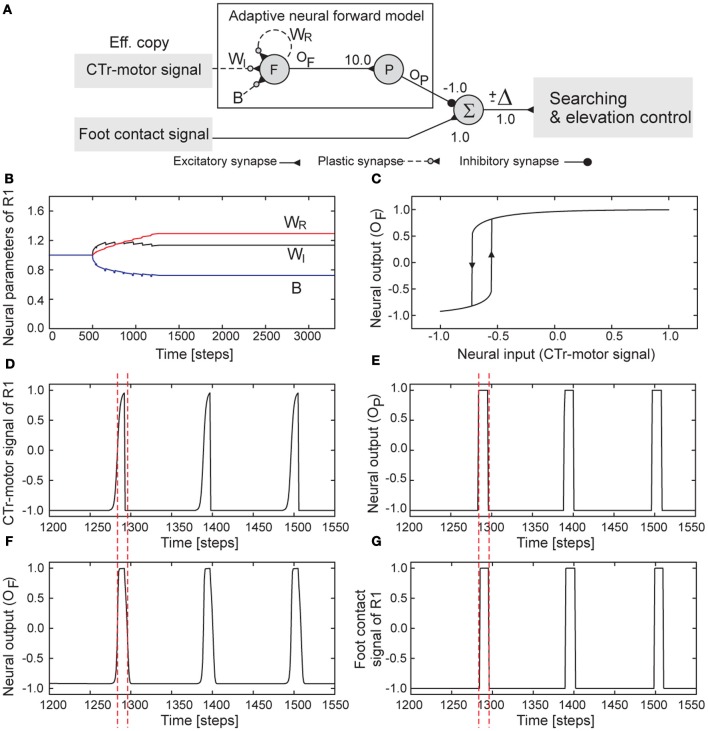
**Adaptive neural forward model. (A)** The model structure consisting of recurrent and non-recurrent neurons. **(B)** Changes of the parameters of the model of the right front leg (R1). **(C)** The hysteresis effect between the input and output signals of the forward model of R1 where the converged parameters are used (see **B**). In this situation, the input varies between −1.0 and 1.0. Consequently, the output will gradually show high activation (≈ + 1.0) when the input increases to value above −0.55. The output will show low activation (≈ −1.0) when the input decreases below −0.715. **(D)** The CTr-motor signal of R1 which is the input of the neuron *F*. Its high activation drives the leg to swing (i.e., swing phase) while its low activation drives the leg in touching the ground (i.e., stance phase). **(E)** The output of the postprocessing neuron *P* is used to compare to the foot contact signal for estimating the walking state. **(F)** The output of the neuron *F* or the transformed motor signal. **(G)** The foot contact signal of R1. It is filtered and mapped onto the interval [−1,+1] where +1 is the leg has no ground contact and vice versa. Dashed lines are provided for comparison. Note that the parameter changes of the forward models of the other legs show similar patterns. Their convergence was achieved after about eight to twenty walking steps. The parameters converged at slightly different values, resulting in slightly different hysteresis loops. One time step is ≈0.037 s.

The adaptive neural forward model serves to estimate the walking state. To do so, it transforms a motor signal (i.e., here the CTr-motor signal^4^, efference copy) into an expected sensory signal to be able to compare it to the actual incoming one (i.e., here the FC signal of the leg). The forward model consists of only two neurons (Figure 6A). The neuron *F* transforms the motor signal while the neuron *P* performs postprocessing. We construct the neuron *F* as a hysteresis element (Pasemann, 1993) using a single recurrent neuron with synaptic plasticity (described below in details) and the postprocessing neuron *P* as a standard one (see Equation 1) with a tanh transfer function. Note that this postprocessing neuron *P* with its large fixed presynaptic weight (i.e., 10.0) basically sharpens a transformed motor signal to perfectly match to a FC signal.

Due to a delay in the relation between FC signal and the CTr-motor signal, a simple thresholding method cannot be applied for signal transformation. Therefore, we use the single recurrent neuron instead since this is a simple neural mechanism providing dynamical properties (e.g., hysteresis effect) that can smooth the motor signal and at the same time provide a delay in the input–output relation required to transform the motor signal into the expected sensory signal. The activation function of this neuron is given by:

(5) $$a_{F}(t)=W_{R}(t)o_{F}(t-1)+W_{I}(t)I(t)+B(t),$$

where *I* is the input of the neuron which is here the CTr-motor signal coming from the CPG-based control. *o*_*F*_ is the output of the neuron given by the tanh transfer function, i.e., *o*_*F*_ = tanh(*a*_*F*_), ∈ [−1, 1]. *W*_*R*_, *W*_*I*_, and *B* are the recurrent weight, the presynaptic weight, and the bias term of the neuron, respectively. These parameters need to be adjusted to obtain a proper hysteresis loop for the signal transformation. Therefore, we employ a gradient descent learning rule to adapt them. In principle, the rule attempts to minimize the error *E* between the target output *T* and the actual output *o*_*F*_ of the neuron through gradient descent. The error is measured as:

(6) $$E(t)=\frac{1}{2}{\left(T(t)-o_{F}(t)\right)}^{2}.$$

In this study, we use the filtered FC sensor signal, linearly mapped onto the interval [−1, 1], as the target output. According to the learning rule, the parameters (*W*_*R*_, *W*_*I*_, and *B*) are updated every time step (≈0.037 s) in proportion to the gradient and given as follows:

(7) $$\Delta W_{R}=-\mu \frac{\partial E}{\partial W_{R}}=\mu (T(t)-o_{F}(t))(1-o_{F}{\left(t\right)}^{2})o_{F}(t-1),$$

(8) $$\Delta W_{I}=-\mu \frac{\partial E}{\partial W_{I}}=\mu (T(t)-o_{F}(t))(1-o_{F}{\left(t\right)}^{2})I(t),$$

(9) $$\Delta B=-\mu \frac{\partial E}{\partial B}=\mu (T(t)-o_{F}(t))(1-o_{F}{\left(t\right)}^{2}),$$

where μ is the learning rate which is set to a small positive value, e.g., 0.01. For the training process, we initialize the neural activity and output states of the forward model to 0.0 and *W*_*R*_, *W*_*I*_, and *B* to 1.0. Due to this simple neural system, the process can perform online. We implemented six forward models on AMOS II where each of them works on one leg. Afterwards, we let AMOS II walk in a normal condition (i.e., walking on floor with a certain gait). The training process will stop as soon as the difference between the filtered FC signal and the postprocessed neural output *o*_*P*_ is smaller than a threshold, e.g., 0.05, over a certain period of times (e.g., 500 time steps). We performed the training process only once and only for the normal walking condition. This walking condition is used as a reference to compare it to other walking conditions in any terrain.

Figure 6B illustrates the parameter changes of the forward model of, e.g., the right front leg (R1, Figure 1A) during training. The training process was set to start after 500 time steps (or around four walking steps) and the parameters (*W*_*R*_, *W*_*I*_, and *B*) converged after around 1300 steps (or around seven walking steps). The resulting parameters lead to a proper hysteresis loop (Figure 6C). Utilizing this hysteresis property together with the neural postprocessing, the CTr-motor signal is finally transformed into the expected FC signal (Figures 6D–G). In this example, AMOS II walked with a slow wave gait (i.e., *MI* = 0.02). It is important to note that the models of all legs that adapted to this gait can be directly applied to other gaits.

**Figure 7:**
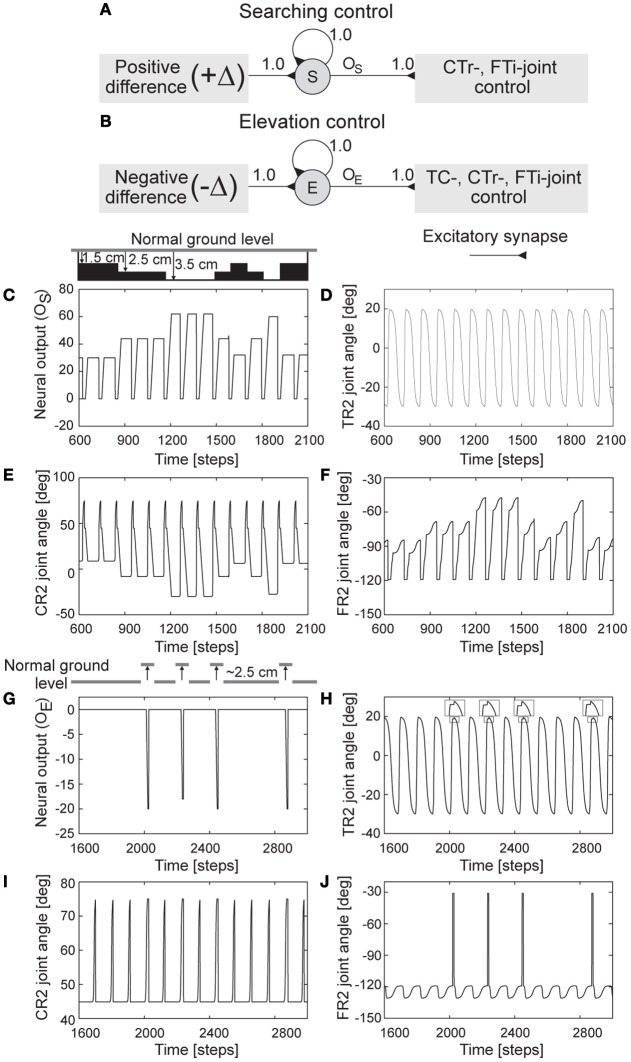
**Searching and elevation control. (A,B)** The neural structures of searching and elevation control. **(C)** Neural output *o*_*S*_ of the searching control, i.e., an accumulated positive error. **(D–F)** The real-time data of the TC-, CTr-, and FTi-joint angles of the right middle leg (R2) showing foothold searching. The drawing above **(C)** shows different generated ground levels (1.5, 2.5, and 3.5 cm below normal ground level) activating foothold searching. **(G)** Neural output *o*_*E*_ of the elevation control, i.e., an accumulated negative error. **(H–J)** The real-time data of the TC-, CTr-, and FTi-joint angles of R2 showing normal leg motion and elevation. In these experiments, the leg is driven by low frequency CPG signals (i.e., *MI* of the CPG is set to 0.02). The drawing above **(G)** shows a generated ground height (≈2.5 cm above normal ground level) activating leg elevation. One time step is ≈0.037 s.

After training, the output of each trained forward model (i.e., the expected FC signal, Figure 6E) is used to compare it to the actual incoming FC signal of the leg (Figure 6G). The difference Δ (Figure 6A) between them determines the walking state where a positive value (+Δ) means losing ground contact during the stance phase and a negative one (−Δ) means stepping on or hitting obstacles during the swing phase. Thus, we use the positive value for searching control (Figure 7A). The value is accumulated through a recurrent neuron *S* with a linear transfer function and always reset to 0.0 at the beginning of swing phase. The output of this neuron *o*_*S*_ with significant change (e.g., *o*_*S*_ > 0.15) controls vertical shifting of the CTr- and FTi-joints. Consequently, these joints are shifted when the positive difference occurs; thereby, the respective leg searches for a foothold. This searching control only occurs in the stance phase. On the other hand, we use the negative value for elevation control (Figure 7B). The value is also accumulated through a recurrent neuron *E* with a linear transfer function. The output of this neuron with significant change^5^ (e.g., *o*_*E*_ < −15) shifts the CTr- and FTi-joint movements upwards. At the same time, the TC-joint movement is shortly inhibited. As a consequence, the leg is elevated, thereby avoiding an obstacle or freeing itself from the obstacle. This elevation control only occurs in the swing phase. Note that the IR sensors installed at the legs (Figure 1B) can be also used for elevation control. This allows the legs to avoid hitting a large obstacle in the front (**Video S5**).

To illustrate the functionality of the searching control and clearly observe leg motion, we activated one leg [e.g., right middle leg (R2)] and fixed the other legs to a certain position. Afterwards, we changed ground level during stance phase. Changing it causes different positive errors (+Δ) due to mismatch between the expected FC signal and the actual incoming one. The error is accumulated through the recurrent neuron *S*. If the accumulated error (Figure 7C) is higher than the threshold, the searching controller then controls the CTr- and FTi-joints to depress the leg and at the same time extend the tibia, respectively. This results in searching for a foothold. Note that the TC-joint motion is not influenced. All joint angles of the leg in this experiment are shown in (Figures 7D–F). We encourage readers to also see the video of this experiment at **Video S6**.

To illustrate the functionality of the elevation control and clearly observe leg motion, we also activated only one leg [e.g., right middle leg (R2)] and fixed the other legs to a certain position. In addition, we inhibited the searching control such that the leg could not search for a foothold. This is to better see and understand the changes of the joint angles. To force elevation of the leg, we made the foot touch an obstacle during the swing phase. This causes negative errors (−Δ) that are accumulated through the recurrent neuron *E*. If the accumulated error (Figure 7G) is higher than the threshold, the elevation controller then inhibits the TC-joint for the forward motion of the leg and at the same time drives the CTr- and FTi-joints to elevate the leg and fully extend the tibia, respectively. This results in the elevation of the leg, thereby freeing it from the obstacle during the swing phase. After the leg frees from the obstacle, the TC-, CTr-, and FTi-joints immediately return to their unaltered positions. Since the process occurs in a very short time, the gait does not break down (see the section 3). All joint angles of the leg in this experiment are shown in (Figures 7H–J). We encourage readers to also see the video of this experiment at **Video S5**.

## 3. Results

In the previous sections, we showed the individual functionalities and performances of the CPG-based control and the local leg control in part. Here, we present experiments carried out to assess the ability of their combination (i.e., adaptive neural locomotion control, Figure 2). The first experiment investigated energy-efficient gaits for different terrains. To do so, we categorized terrains into four different groups: hard terrain (e.g., floor, pavement), loose terrain (e.g., fine gravel), rough terrain (e.g., gravel), and vegetated terrain (e.g., grass).

**Figure 8:**
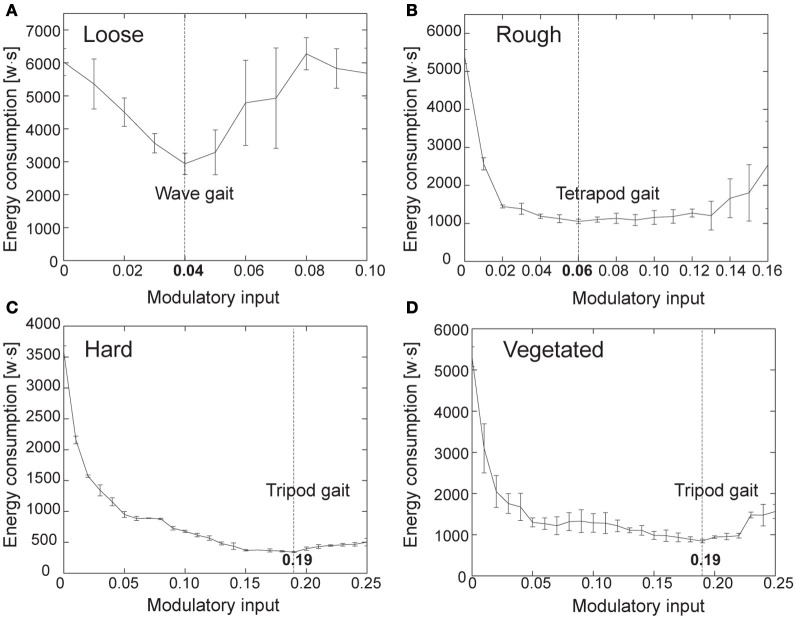
**Electric energy consumptions for different terrain groups and gaits. (A)** Loose terrain. **(B)** Rough terrain. **(C)** Hard terrain. **(D)** Vegetated terrain. Each measurement was repeated five times. Dashed line of each plot indicates the *MI* value for energy-efficient locomotion.

For each of these terrain groups, we let AMOS II walk from slow to fast gaits by manually increasing *MI* of the CPG. During locomotion, the local leg control autonomously adapted the legs for a foothold. Thus, in this experiment, the CPG-based control and the local leg control function as open-loop control and closed-loop control, respectively. We calculate the electric energy consumption of each walking pattern as:

(10) E=IVt,

where *I* is average electric current in amperes used by the motors during walking 1 m. It is measured using the Zap 25 CS installed inside AMOS II. *V* is voltage (here 5 V). *t* is time in seconds for the travel distance (here 1 m). Figure 8 shows the energy consumptions measured in these four terrain groups where the measurement of each group was repeated five times.

Figures 8A,B suggest using the *MI* values of 0.04 and 0.06 which generate a fast wave gait and a tetrapod gait on loose and rough terrains, respectively. Figures 8C,D suggest using the *MI* value of 0.19 which produces a fast tripod gait on hard and vegetated terrains. Note that AMOS II started to slip when the value of *MI* was higher than 0.19 for hard and vegetated terrains and it got stuck most of the time when the *MI* values were higher than 0.16 and 0.10 for rough and loose terrains, respectively. This experimental result reveals that each terrain group requires a specific gait which leads to the lowest energy consumption. This allows mapping the four terrain groups to the energy-efficient gaits.

**Figure 9:**
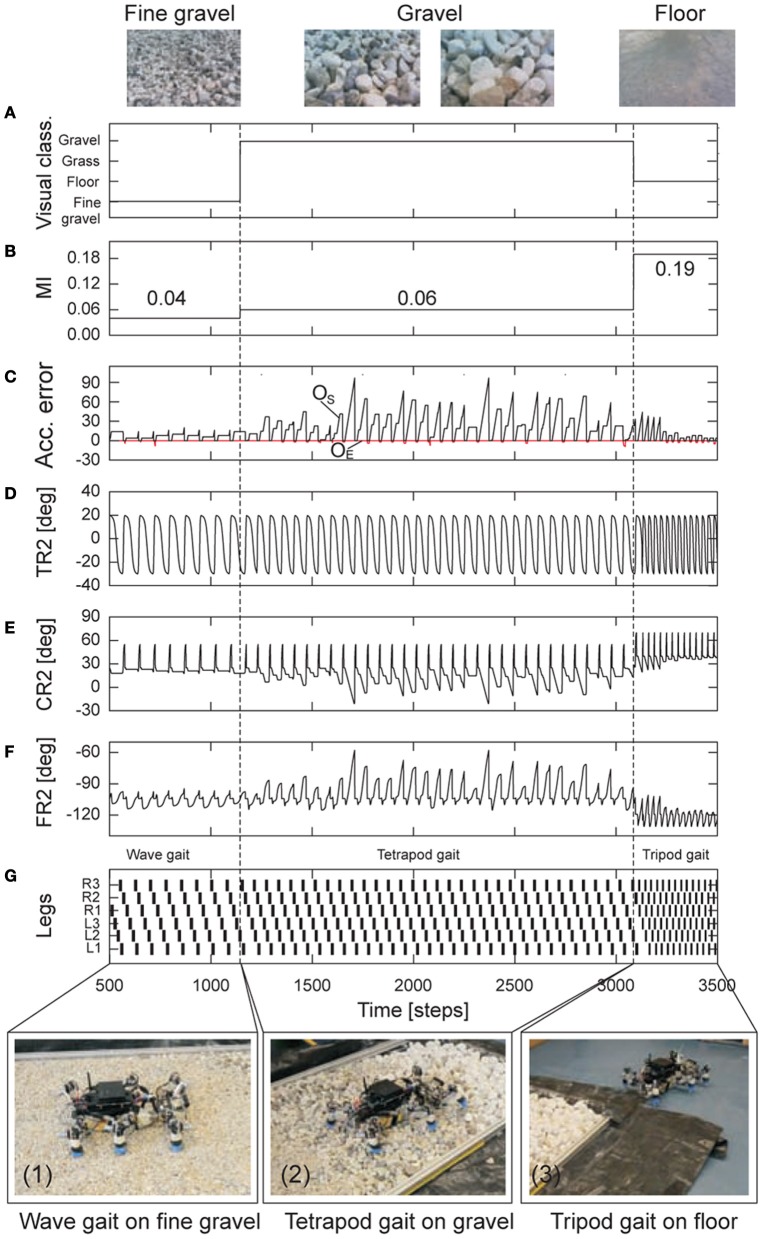
**Real-time data of energy-efficient and adaptable locomotion on three different terrains. (A)** The output of the online terrain classification system which is a preprocessed visual sensory signal. **(B)** The modulatory input *MI* of the CPG which is directly controlled by the sensory signal. It was set to 0.04 (fast wave gait), then 0.06 (tetrapod gait), and finally 0.19 (fast tripod gait). **(C)** The positive (*o*_*S*_) and negative (*o*_*E*_) accumulated errors (Figures 7A,B). They control leg adaptation to deal with different terrains. **(D–F)** The TC-, CTr-, and FTi-joint angles of the right middle leg (R2) during walking from fine gravel (loose terrain) to gravel (rough terrain) to floor (hard terrain). They represent the leg movement including adaptation. **(G)** Gait diagram showing the different energy-efficient gaits of AMOS II while traversing the terrains. Black boxes indicate swing phase while white areas between them indicate stance phase. Abbreviations are referred to Figure 1. Above pictures show snap shots from the camera on AMOS II used for the terrain classification while walking. Below pictures show snap shots of locomotion of AMOS II during the experiment. Note that one time step is ≈ 0.037 s.

The second experiment employed the investigated energy-efficient gaits together with the visual terrain classification system (described in section 2.3) to allow AMOS II to autonomously perform energy-efficient locomotion while traversing the different terrains. The output of the visual terrain classification system provides terrain information. This information was used as the preprocessed sensory input to set *MI* of the CPG, thereby triggering the corresponding pre-mapped energy-efficient gait. This way, the experiment reflects a complete neural closed-loop system (Figure 2). The experimental result is shown in Figure 9.

**Figure A4:**
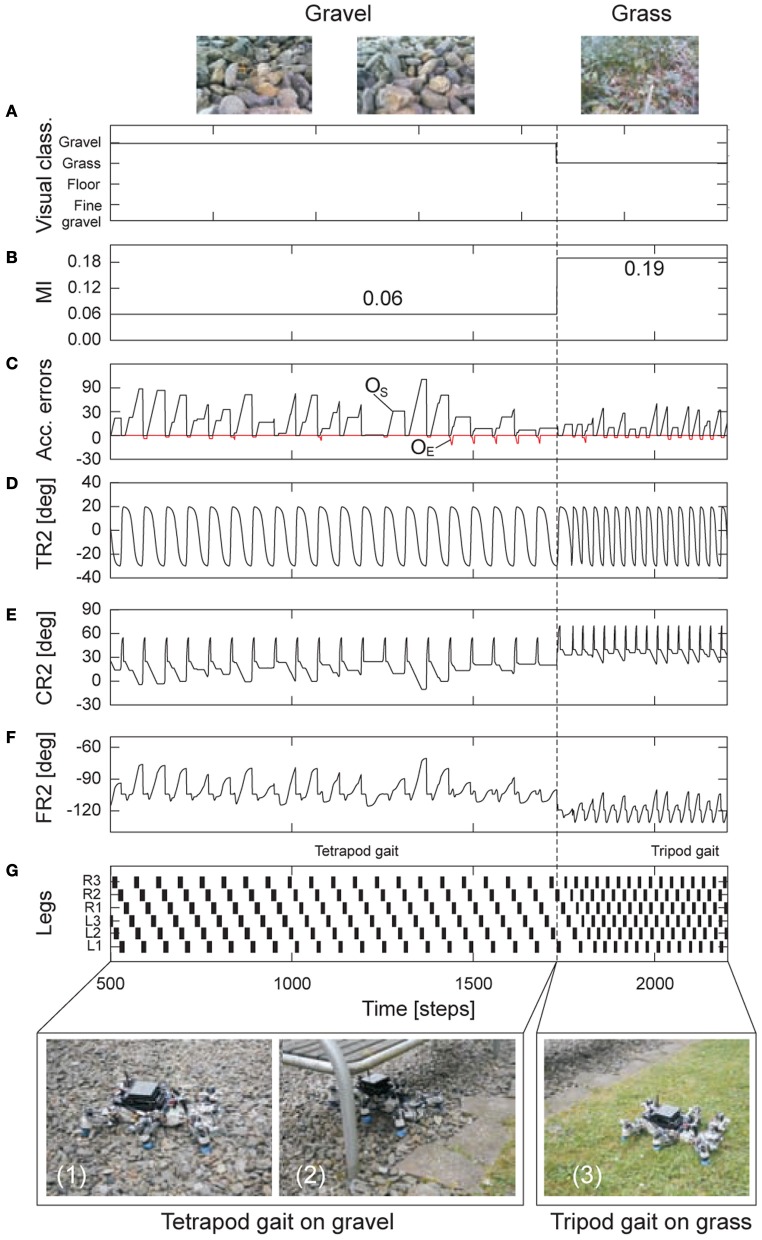
**Real-time data for energy-efficient and adaptable locomotion on two different terrains in an outdoor environment. (A)** The output of the online terrain classification which is a preprocessed visual sensory signal. **(B)** The modulatory input *MI* of the CPG which is directly controlled by the sensory signal. It was set to 0.06 (tetrapod gait) and then 0.19 (fast tripod gait). **(C)** The positive (*o*_*S*_) and negative (*o*_*E*_) accumulated errors of the expected foot contact signal and the actual one (cf. Figures 7A,B of the manuscript). They control leg adaptation to deal with different terrains. **(D–F)** The TC-, CTr-, and FTi-joint angles of the right middle leg (R2) during walking from gravel (rough terrain) to grass (vegetated terrain). They represent the leg movement including adaptation. **(G)** Gait diagram showing the different energy-efficient gaits of AMOS II while traversing the terrains. Black boxes indicate swing phase while white areas between them indicate stance phase. Abbreviations are referred to Figure 1 of the manuscript. Above pictures show snap shots from the camera on AMOS II used for the terrain classification while walking. Below pictures show snap shots of locomotion of AMOS II during the experiment. Note that one time step is ≈0.037 s.

It can be seen that at the beginning AMOS II walked with a fast wave gait (photo 1) since it detected fine gravel (loose terrain) using its visual system. Afterwards, it changed from the wave gait to a tetrapod gait (photo 2) since it detected gravel (rough terrain). Finally, it used a fast tripod gait (photo 3) on the floor (hard terrain). During traversing the different terrains, AMOS II adapted its legs individually to deal with a change of terrain. That is, it depressed its leg and extended its tibia to search for a foothold when losing a ground contact during the stance phase. Losing ground contact information is detected by a significant change of the positive accumulated error *o*_*S*_, see black line in Figure 9C). However, during the swing phase no leg elevation was observed (i.e., no significant change of the negative accumulated error *o*_*E*_, see red line in Figure 9C) since only minor perturbation occurred, where the perturbation was handled by the passive components of the leg. We encourage readers to see the video of this experiment at **Video S7**. Another test in an outdoor environment where AMOS II walked from gravel to grass can be seen at Figure A4. In addition to energy-efficient and adaptable locomotion emphasized in this experiment, the basic leg movements of AMOS II and the used gait follows insect locomotion. Thus, this experiment is an example of the demonstration of insect-like, energy-efficient, and adaptable locomotion of walking machines, like AMOS II.

**Figure 10:**
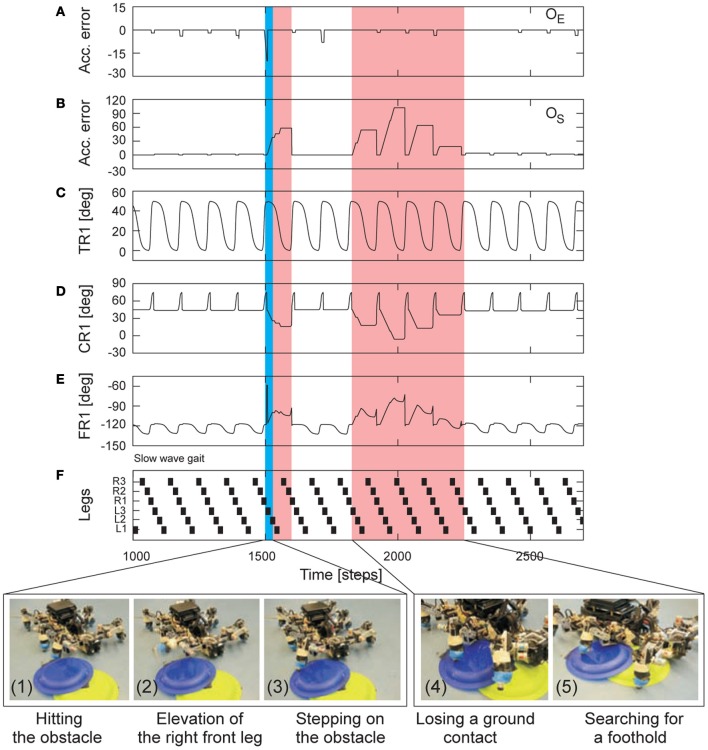
**Real-time data of adaptable locomotion on terrain with small obstacles. (A,B)** The negative (*o*_*E*_) and positive (*o*_*S*_) accumulated errors (Figures 7B,A). They control leg adaptation to deal with stepping on or hitting obstacles during the swing phase and losing a ground contact during the stance phase. **(C–E)** The TC-, CTr-, and FTi-joint angles of the right front leg (R1) during walking on the floor with small obstacles (≈2.5 cm height). They represent the leg movement including adaptation. **(F)** Gait diagram showing a slow wave gait (*MI* = 0.02) of AMOS II in this experiment. Black boxes indicate swing phase while white areas between them indicate stance phase. Abbreviations are referred to Figure 1. Below pictures show snap shots of locomotion of AMOS II during the experiment. Blue and red areas indicate elevation and searching actions, respectively. Note that one time step is ≈0.037 s.

The third experiment focused on both, leg elevation and foothold searching, of AMOS II to deal with small obstacles. In this scenario, we let AMOS II walk with a certain pattern [e.g., a slow wave gait (*MI* = 0.02)] and placed small obstacles (≈2.5 cm height) on its path. The experimental result is shown in Figure 10. It can be seen that, while walking forward, the foot of the right front leg (R1) of AMOS II hit an obstacle during the swing phase (photo 1), thereby preventing the leg from completing the phase. This leads to a significant change of the negative accumulated error *o*_*E*_ (Figure 10A). As a consequence, AMOS II elevated the leg to free it from the obstacle (photo 2). Afterwards, it placed the leg on top of the obstacle without getting stuck (photo 3). Due to the difference of the ground level, this causes a significant change of the positive accumulated error *o*_*S*_ (Figure 10B). AMOS II then lowered the leg more downward to ensure ground contact. After a few steps, the leg again lost a ground contact during the stance phase (photo 4), resulting in searching for a foothold (photo 5). Finally, AMOS II successfully walked away from the obstacles. This experiment reveals that using this leg adaptation mechanism AMOS II can effectively locomote on terrain with small obstacles without getting stuck. We encourage readers to also see the video of this experiment at **Video S8**.

**Figure 11:**
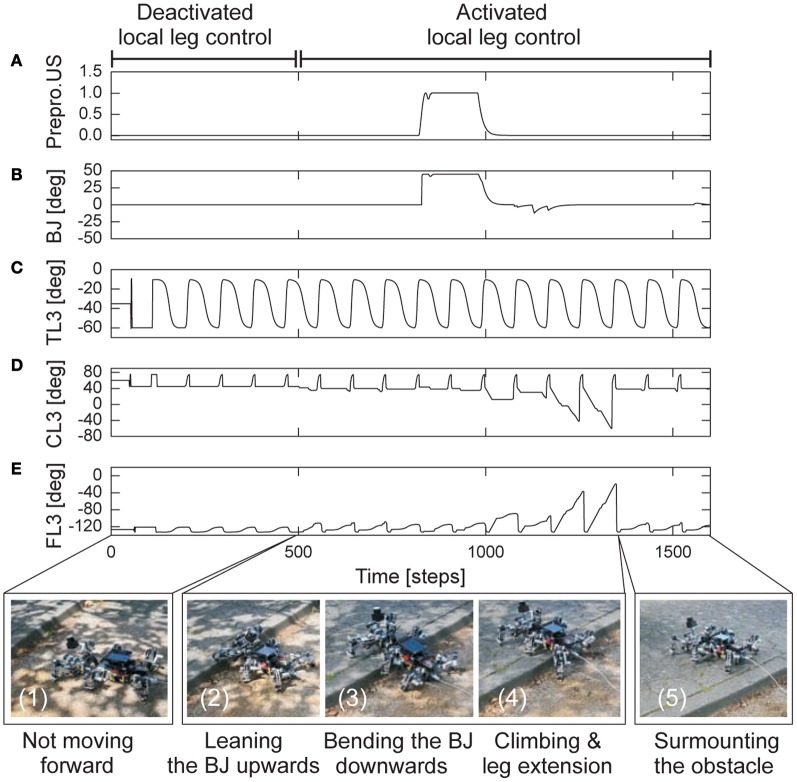
**Real-time data of walking and climbing over a large obstacle in an outdoor environment. (A)** The preprocessed ultrasonic sensor (US) signal for reactive backbone joint control. **(B)** The backbone joint (BJ) angle during walking and climbing. The BJ stayed at zero angle during walking. It leant upwards and then bent downwards during climbing. **(C–E)** The TC-, CTr-, and FTi-joint angles of the left hind leg (L3) during walking and climbing. The joint adaptation was controlled by the negative (*o*_*E*_) and positive (*o*_*S*_) accumulated errors (Figures 7B,A). The changes of the errors have similar patterns as shown in Figure 9C. Here AMOS II used a slow wave gait (*MI* = 0.02, Figure 10F). Below pictures show snap shots of the locomotion of AMOS II during the experiment. Note that one time step is ≈0.037 s.

The fourth experiment was to show that the adaptive neural locomotion control not only generates insect-like, energy-efficient, and adaptable locomotion of AMOS II (as shown above) but also allows it with the help of its BJ to climb over a large obstacle. To do so, we placed AMOS II on rough terrain (i.e., soil with stones) with an 11 cm high obstacle at front. The task of AMOS II was to move forward and climb over the obstacle. For this experiment, the CPG-based control generated a basic walking pattern [e.g., a slow wave gait (*MI* = 0.02)] while the local leg control adapted the legs individually for foothold searching and elevation, thereby enabling effective locomotion and supporting the body of AMOS II during climbing. Note that the slow wave gait was used in this experiment because it is the most effective gait for climbing which allows AMOS II to negotiate the highest climbable obstacle (13 cm height which equals 75% of its leg length) [see Goldschmidt et al. (2012) for details]. In addition to the locomotion control, reactive BJ control was also applied to control the BJ for climbing [see Goldschmidt et al. (2012) for details]. The controller produces an abstraction of body flexion observed in cockroach climbing. It controls the BJ to lean upwards to surmount obstacles and to bend downwards for stable climbing. This downward motion appears in cockroach climbing while the upward motion does not exist. Instead of leaning the body flexion joint upwards as AMOS II does, a cockroach extends its front and middle legs to raise its reaching height to surmount obstacles, thereby rearing its entire body to a taller pose. Here, we used the US at the front body part of AMOS II (Figure 1A) for obstacle detection and BJ control. Figure 11 presents the experimental result.

At the first period (0–500 time steps), the local leg control was deactivated. Due to the rough terrain, the feet could not perfectly touch the ground during the stance phase; thus, AMOS II could not move forward (photo 1). After 500 time steps, the local leg control was activated. It allows for foothold searching, thereby adapting locomotion to the terrain. As a result, AMOS II moved forward. As AMOS II approached the obstacle, the US detection activated the BJ control such that the BJ leant upwards (photo 2). Due to a time-out period after leaning upwards, the BJ moved downwards to ensure stability while climbing (photo 3). During climbing, a hind leg [e.g., left hind leg (L3), photo 4] lowered downwards, showing leg extension, to support the body. Finally, AMOS II successfully locomoted on rough terrain and surmounted the 11 cm high obstacle (photo 5). We encourage readers to also see the video of this experiment at **Video S9**. Besides this experimental result, it is important to note that both adaptive locomotion and reactive BJ controllers have a distributed implementation, but they are indirectly coupled by sensory feedback and the physical components of AMOS II. This way, the combined neural control network driven by the sensor signals synchronizes leg and BJ movements for stable walking and climbing.

**Figure 12:**
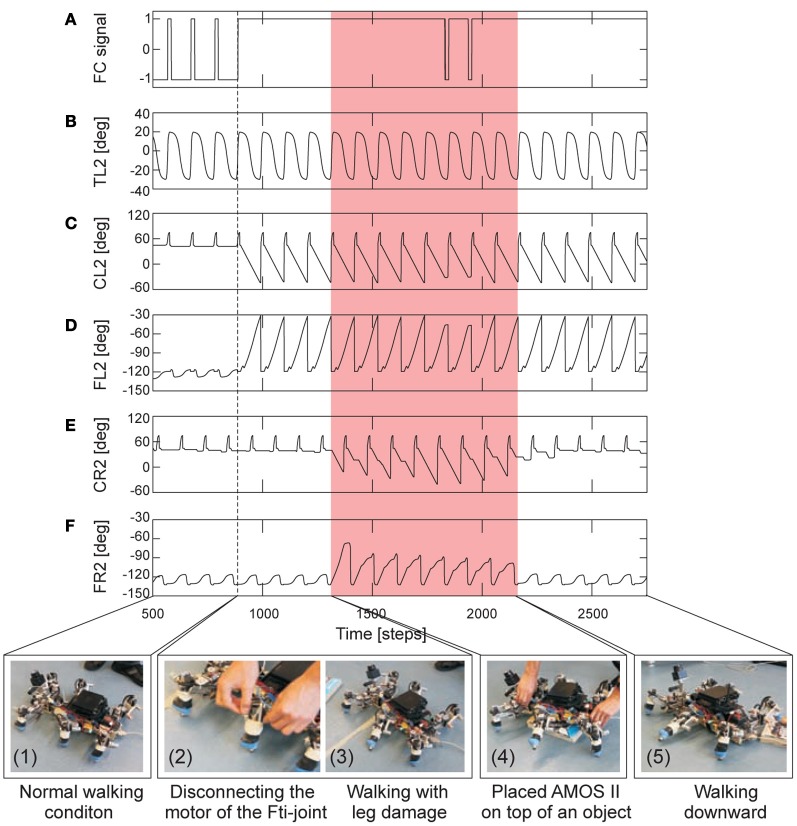
**Real-time data of adaptable locomotion during leg damage. (A)** The filtered foot contact (FC) signal of the left middle leg (L2) where +1 is the leg has no ground contact and −1 is the leg touches the ground. **(B–D)** The TC-, CTr-, and FTi-joint angles of L2. **(E,F)** The CTr- and FTi-joint angles of the right middle leg (R2). The joint adaptation was controlled by the negative (*o*_*E*_) and positive (*o*_*S*_) accumulated errors (Figures 7B,A). The changes of the errors have similar patterns as shown in Figure 9C. Here AMOS II used a slow wave gait (*MI* = 0.02, Figure 10F). Below pictures show snap shots of the locomotion of AMOS II during the experiment. Dashed line indicates the time that the motor power connector of the FTi-joint of L2 was disconnected. Red area indicates the time that AMOS II was on a 3.5 cm high object. Note that one time step is ≈0.037 s.

The final experiment was to illustrate that the adaptive neural locomotion controller can adapt the remaining legs to deal with a leg damage situation. In this experiment we let AMOS II walk with a slow wave gait (*MI* = 0.02) and then disconnected the power connector of the motor of a leg joint such that the joint became inactive (i.e., uncontrollable). This is to simulate leg damage. After damage, we placed AMOS II on top of an object to observe the adaptation of the remaining legs that allows AMOS II to be able to continue moving forward. Figure 12 present the experimental result.

As shown in Figure 12, AMOS II walked in a normal walking condition at the beginning (photo 1). During walking, we disconnected the motor power connector of the FTi-joint of the left middle leg (photo 2) such that the joint became inactive. Then we also tilted the tibia upward; thereby, the foot could not touch the ground properly. This results in the leg adaptation to search for a foothold (photo 3). Afterwards, we placed AMOS II on top of a 3.5 cm high object (photo 4). Since AMOS II was on the object, its legs lost a ground contact. AMOS II adapted its legs to search for a foothold (see, e.g., the FTi- and CTr-joint signals of the right middle leg in Figures 12E,F). As a result, it successfully climbed down from the object and continued walking forward (photo 5). The ability of leg adaptation was mainly achieved by the local leg control mechanisms. These mechanisms even allow AMOS II to climb down from the object with a 7 cm height. Without them, AMOS II got stuck on the object. We encourage readers to see the video of this experiment at **Video S10**. This experimental result reveals that the developed adaptive neural locomotion controller can not only generate versatile locomotion behaviors including climbing (shown in the other experiments) but also give robustness to the system by allowing it to cope with damage.

## 4. Discussion

Here, we briefly discuss some remaining issues concerning the six-legged walking machine AMOS II and its controller, because most of the relevant discussion points have been treated in the above sections.

AMOS II was used as an experimental platform and represents an embodied neural closed-loop system with many degrees of freedom. It was designed with a morphology analogous to a cockroach. It was constructed in a straightforward way as a biomechatronic system consisting of several sensors and actuators. Due to extra rubber coupling elements and springs integrated into the joints and tibiae of AMOS II, this yields passive compliance allowing AMOS II to deal with minor disturbances during locomotion over rough terrain (as described in the second experiment). The joint compliance also enables AMOS II to passively flex its legs to avoid damages when the environment changes (**Video S11**). Besides the physical components of AMOS II that follow biomechanics of walking animals, another special trait of AMOS II is that we configured the ranges of the joint movements of AMOS II such that it has a very low center of mass (i.e., low ground clearance) and its body falls to the ground before taking the next step during normal walking. When negotiating a large obstacle, AMOS II uses its BJ together with additional reactive BJ control (Goldschmidt et al., 2012) to climb over it while its leg movements automatically adapt accordingly (**Video S12**).

**Figure A5:**
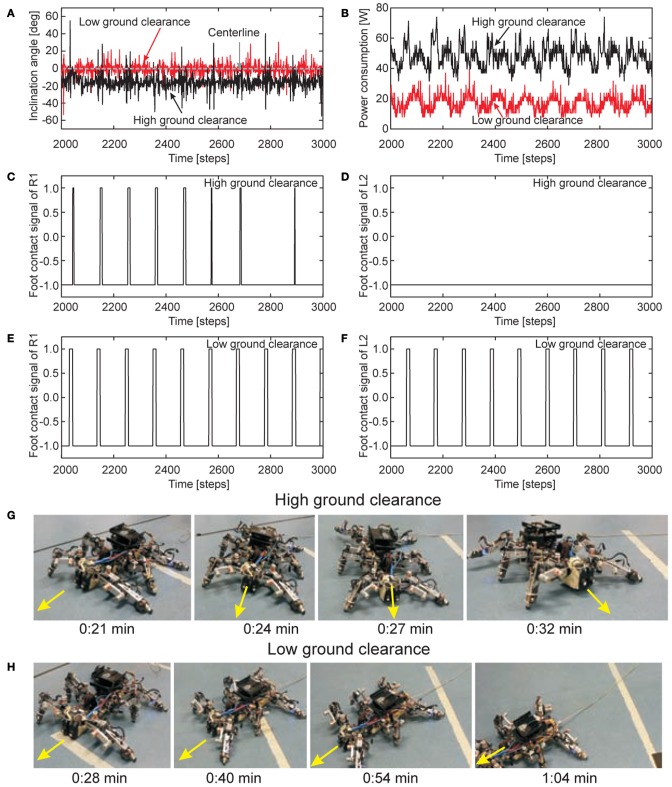
**Real-time data for walking with high and low ground clearance during leg damage. (A)** The inclination angle of AMOS II obtained from an inclinometer sensor installed inside the body. Negative value means that AMOS II tilts to its left. **(B)** Power consumption. **(C,D)** The foot contact signals of R1 and L2 for the high ground clearance case. **(E,F)** The foot contact signals of R1 and L2 for the low ground clearance case. They are filtered and mapped onto the interval [−1, +1] where +1 is the leg has no ground contact and vice versa. **(G,H)** Snap shots of the locomotion of AMOS II during the test with high and low ground clearance, respectively.

In fact, the advantage of low ground clearance is evident in case of leg damage. In this situation, a robot with high ground clearance will tip over or fall down a lot (Figure A5A) leading to unstable locomotion and remaining legs need to carry more load. Thus, the motors need to produce high torque to carry the load resulting in high power consumption (Figure A5B). Furthermore the legs might have difficulty to swing during swing phase (Figures A5C, D); thereby, the robot will not move forwards properly (Figure A5G and **Video S10**). In contrast, with low ground clearance the robot will not much fall down (Figure A5A) since its body is already close to the ground and the remaining legs need not to carry much more load leading to lower power consumption compared to the high ground clearance case (Figure A5B), and they are able to swing during swing phase (Figures A5E, F). As a result, the robot can still move better in a straight way (Figure A5H and **Video S10**). However, the drawback of having low ground clearance is that the robot could get stuck often when walking on non-flat terrains. Accordingly, during walking over rough terrains AMOS II will lift its body up to obtain higher ground clearance such that it does not get stuck. Lifting the body up is automatically done by shifting the center of the CTr-joint angles downwards (more depression) and the center of the FTi-joint angles upwards (more extension) and this is the default joint movements for rough terrains. By contrast, most walking machines (Lee et al., 2006; Spenneberg and Kirchner, 2007; Lewinger and Quinn, 2009) always perform locomotion with high ground clearance (**Video S12**). Although such a high ground clearance walking strategy could simplify it for the controller to deal with different terrains, it might lead to instability of the systems (as described above); unless, additional control mechanisms are applied (Spenneberg et al., 2004). In fact, the biologically-inspired locomotion strategy of AMOS II arises not only from biomechanics but is a combination of its biomechanics and adaptive neural locomotion control. While the biomechanics allows for leg and body movements as well as provides some degree of disturbance rejection, the adaptive neural locomotion controller generates versatile motions and adaptation.

The controller consists of two main parts: CPG-based control and local leg control. The CPG-based control is the improved version of our original chaotic CPG-based controller [compare Figure A2 in Steingrube et al. (2010) with Figure A2 of this paper]. Two main components of the controller have been modified here while the other parts remain unchanged. We replaced the chaotic CPG by a simpler CPG mechanism with neuromodulation. As a consequence, by exploiting neural dynamics of the new CPG mechanism, we can generate a multitude of walking patterns (e.g., 20 patterns). Some of these patterns are comparable to insect gaits (Wilson, 1966) and allow for energy-efficient locomotion on different terrains, like, fine gravel (loose terrain), gravel (rough terrain), and grass (vegetated terrain). The CPG also provides fast switching between the patterns compared with the chaotic CPG. For motor connections, we modified the connections to the FTi-motor neurons such that the FTi-joints are activated during walking while in the previous work these joints are inhibited; i.e., they stay in a flexed position. The introduced FTi-joint movements are inspired by insect leg movements (Ekeberg et al., 2004; Cruse et al., 2009). During the stance phase of forward walking, the FTi-joints of the front legs flex inward, of the hind legs extend outward, and of the middle legs combine these two movements by first flexion and then extension. As a consequence, the front, hind, and middle legs pull, push, and pull and push the body forward, respectively. This results in faster walking speed compared with the fixed FTi-joint version. This CPG-based control coordinating all joints can be considered as open-loop control since in principle it does not require any sensory feedback for the locomotion generation (i.e., multiple patterns and insect-like leg movements). However, the loop can be simply closed by using, e.g., exteroceptive sensory feedback to generate stimulus induced behavior (like, photo tropism and obstacle avoidance) as well as to select an energy-efficient gait with respect to the terrain in an autonomous manner.

In contrast to the CPG-based control, the local leg control introduced here for the first time employs proprioceptive sensory feedback (i.e., here only FC sensors) for adaptable locomotion. Thus it can be considered as closed-loop control. It has two components applied independently to each leg of AMOS II: an adaptive forward model with efference copy and searching and elevation control. The forward model is constructed by using a simple hysteresis neuron with recurrent connection. It can learn online to transform the CTr-motor signal (efference copy) into the expected FC signal. While the forward model is minimal and sufficient here, one could combine several of them to obtain different forward models for different purposes, e.g., sensory noise cancelation and slope detection (Manoonpong and Wörgötter, 2009) or use them for designing non-linear filters (Manoonpong et al., 2010). Due to our controller being modular, if desired, one could replace this simple hysteresis neuron by more complex neural networks [e.g., reservoir computing networks (Dasgupta et al., 2012)] for transforming motor commands into complex expected sensory signals.

Our forward model presented here can be considered as an adaptive predictor that can learn to predict the sensory consequences (expected sensory feedback) from motor commands (efference copy) (Kawato, 1999). The expected sensory feedback (or transformed motor command) is then used to compare it with the actual FC signal for the walking state estimation. The sensory prediction error enables AMOS II to determine whether its leg loses ground contact during the stance phase or hits or steps on any obstacles during the swing phase. Afterwards, this information is used to adapt the leg accordingly through the searching and elevation control. The adaptive leg motions (i.e., searching and elevation motions) follow the observed locomotion in certain insects, like locusts (Pearson and Franklin, 1984), cockroaches (Tryba and Ritzmann, 2000), and stick insects (Fischer et al., 2001), during walking on rough terrain. As a result, employing closed-loop local leg control mechanisms with the forward models allows AMOS II to not only successfully traverse rough terrains and climb over large obstacles, but to also cope with leg damage.

Besides special features described above, our adaptive neural locomotion controller also combines three key aspects found in animal locomotor control: central mechanism (CPGs) (Meyrand et al., 1991; Katz, 1998; Harris-Warrick et al., 2011), sensory feedback (afferent-based control) (Cruse et al., 2009), and internal forward models with efference copies (efferent-based control) (Holst and Mittelstaedt, 1950; Cruse et al., 1998; Bläsing and Cruse, 2004). In particular, our CPG-based control or central mechanism for versatile locomotion generation relies on a CPG mechanism with neuronmodulation that is inspired by the function of neural CPG circuits found in lobsters (Selverston et al., 1993; Pulver and Marder, 2002) and the mollusc *Tritonia diomedea* (Katz et al., 1994). These biological findings suggest that extrinsic and intrinsic neuromodulatory inputs to the CPG circuits can alter the cellular changes and synaptic properties of neurons in the circuits. Thereby, these inputs modify the output of the CPG leading to behavioral flexibility and different locomotion modes. This process can be achieved on the fly resulting in the adaptation of behavior to environmental changes in an ongoing fashion. Our local leg control mechanisms based on sensory feedback (afferent-based control) and adaptive neural forward models with efference copies (efferent-based control) for state estimation and adaptable locomotion follows the evidence of forward model predictions with sensory feedback in the stick insects *Aretaon asperrimus*. It shows that during climbing over very large gaps the stick insects perform an immediate change in the stepping pattern of the legs when losing ground contact at the end of the swing phase (Bläsing and Cruse, 2004). This would reflect an expectation of regular ground contacts. Other results supporting the idea of forward model predictions (Cruse et al., 1998) indicate that, during the swing phase of the stick insects, reactions to obstacles depend on an internal state.

While these three key aspects are essential for locomotion control, some works have taken these aspects into account for developing locomotion control in simulation (Kuo, 2002; Dürr et al., 2003). Only a few have successfully applied it to a real system but with small numbers of inputs and outputs and behavioral restrictions (Lewis and Simo, 2001; Lewis and Bekey, 2002), thereby, reducing the sensor-motor coordination problem substantially. Most studies use a combination of several CPGs and sensory feedback to generate different walking behaviors (Beer et al., 1997; Harischandra et al., 2011) including reflexes (Kimura et al., 2007; Spenneberg and Kirchner, 2007; Lewinger and Quinn, 2011; von Twickel et al., 2012). The reflexes driven by only sensory feedback results in searching and elevation actions when losing ground contact and hitting an obstacle, respectively. However, due to the lack of forward model predictions (internal state) this control approach has difficulties to generate reactions for walking machines to avoid an obstacle when stepping on it during swing phase as the stick insects do. Another interesting approach, like “Walknet” (Cruse et al., 2007), has no central control unit. Instead, it uses a decentralized control architecture with local coordination rules highly depending on different types of proprioceptive sensory feedback, e.g., FC, joint angle, and joint angular velocity signals, to determine an internal state and generate basic locomotion and adaptation. However, this mechanism malfunctions when losing the sensory information, thereby it is less robust.

In contrast to this, our adaptive neural locomotion controller based on a modular structure is robust and has fault tolerance capabilities. Damage to a part of the system can result in a loss of some of the abilities of the system, but, the whole system can still function partially (see the leg damage experiment in Figure 12). Its modules (Figures 2, A2) generally have a simpler structure as compared to the network as a whole. Thus, their functions and dynamics are analyzable by observing the input/output relationship of an individual module (Manoonpong et al., 2007, 2008b). Its individual modules have been used in earlier studies and successfully provided partial solutions to different walking machines (Manoonpong et al., 2007, 2008b). Furthermore, the controller, using a single CPG, sensory feedback, and forward model predictions providing an internal state, can generate a multitude of walking patterns (e.g., 20 walking patterns), insect-like leg movements, energy-efficient locomotion, and adaptable locomotion (like searching and elevation actions including reactions when stepping on an obstacle during swing phase). It can also handle leg damage and even generate cockroach-like climbing behavior (**Video S12**) when additional reactive BJ control is applied (Goldschmidt et al., 2012). The controller can also be simply transferred to another six-legged walking machine having a different morphology but leg lengths with similar proportion to AMOS II. In this case, the internal network structure and parameters of its CPG-based control (Figure 2, left) remain unchanged. We set *MI* of the CPG to 0.15; thereby, the controller generates a tripod gait with a walking frequency of approximately 0.8 Hz for the machine (**Video S13**). Only the maximum and minimum ranges of the joint movements of the legs and the neural parameters of the adaptive forward models (Figure 2, right) are different. The neural parameters are adapted to the new system by using the online learning mechanism (Equations 8–9). In principle, applying the controller to other different walking machines might be necessary to also adjust generated walking frequency (i.e., operating range of *MI* of the CPG). The capability of the controller which combines the key aspects of the biological locomotion systems to achieve a very rich behavioral repertoire in an autonomous fashion, to the best of our knowledge, has not been achieved in other walking machine systems so far.

Taken together this work suggests how a CPG mechanism with neuromodulation, sensory feedback, and internal forward models with efference copies can be used for controlling complex robots. It further confirms that this combination plays an important role for locomotion in biological as well as artificial systems. The results presented here show that the employed embodied neural closed-loop system can be an option for developing robust and adaptable machines, thereby bringing the goal of approaching living creatures in their levels of performance a little bit closer. As the controller is modular, it is flexible and offers the future possibility of integrating joint angle and joint CS signals as feedback together with additional entrainment and reflexive mechanisms (Takemura et al., 2005; Cruse et al., 2009; Nachstedt et al., 2012) to avoid leg slipping which currently occurs when the legs work partially against each other. The controller can also be extended to multiple CPGs (Ren et al., 2012) in order to be able to adjust the frequency of each leg individually for some situations like gap crossing (Bläsing, 2006) or damage compensation (Ren et al., 2012). It even can be combined with other neural modules like short term motor memory (Dasgupta et al., 2012) and muscle models (Xiong et al., 2012). This will enable the robotic system to be capable of navigating in complex environments with a certain degree of memory-guided behaviors and at the same time performing more natural movements with active compliances.

### Conflict of interest statement

The authors declare that the research was conducted in the absence of any commercial or financial relationships that could be construed as a potential conflict of interest.

## Figures and Tables

**Figure A3:**
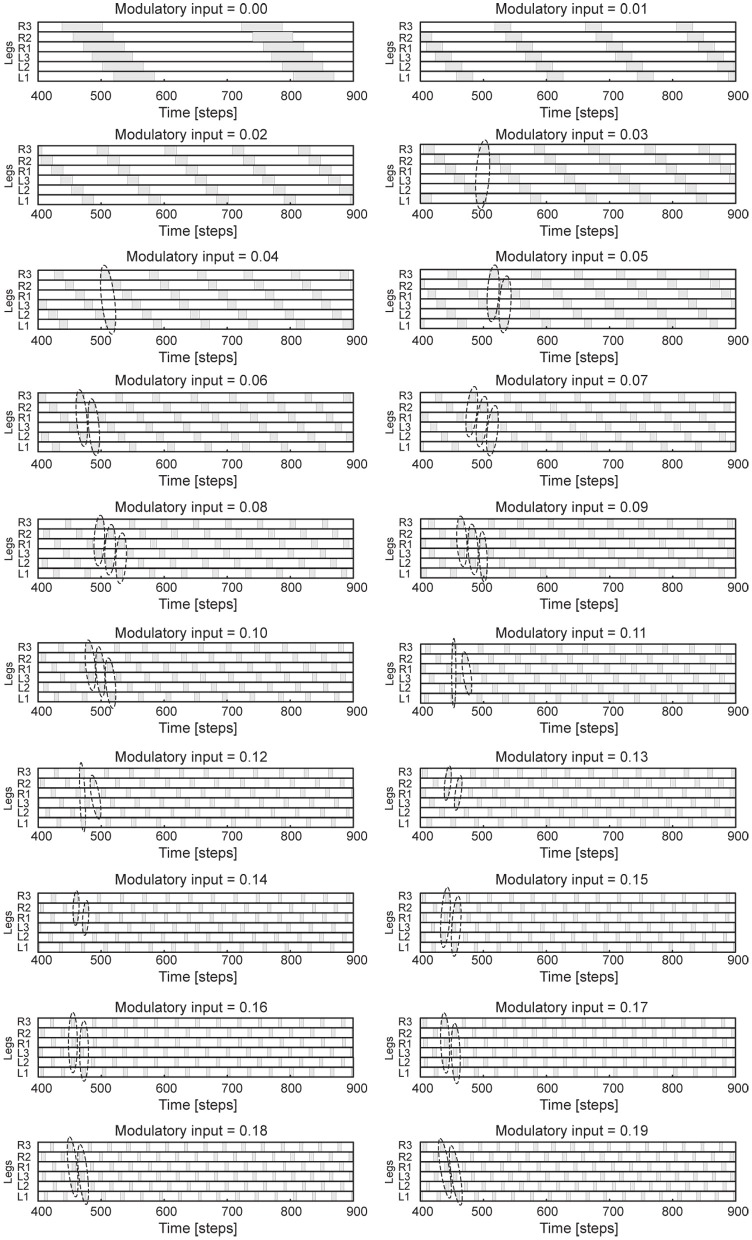
**20 different walking patterns of AMOS II.** The patterns are observed from the motor signals of the CTr-joints. White areas indicate ground contact or stance phase and gray areas refer to no ground contact during swing phase. As frequency increases, some legs steps in pairs (dashed enclosures). One time step is ≈0.037 s.

## Footnotes

^1^Advanced MObility Sensor-driven walking device II.

^2^Note that this limitation is not because of the CPG but due to the hardware. Applying the CPG to different robots (e.g., light weight robots with fast actuator speed), one might be able to obtain more than 20 different walking speeds on flat terrain.

^3^This CPG will show chaotic dynamics if its synaptic weights are set to *W*_11_ = −5.5, *W*_22_ = 0.0, *W*_12_*m*__ = 1.475, *W*_21_*m*__ = −1.65 with additional bias terms (*B*_1_ = −5.725, *B*_2_ = 0.25) projecting to the neurons *C*_1_ and *C*_2_, respectively. The chaotic patterns prove behaviorally useful for self-untrapping from a hole in the ground (Steingrube et al., 2010).

^4^We use the CTr-motor signal instead of the TC- and FTi-motor signals since its pattern is close to the FC signal.

^5^Here, we use a high threshold value for controlling the elevation since a minor disturbance can be handled by passive mechanisms (spring and passive couplings) installed at the leg. Using a small threshold value might lead to an unnecessary elevation of the leg resulting in unstable motion.

## Supplementary material

**Video S1 | Comparison of gait switching using a CPG with neuromodulation and a chaotic CPG.** Using a CPG with neuromodulation, AMOS II shows fast and smooth switching between gaits, while the switching is slower and less smooth when using our previous chaotic CPG. (http://manoonpong.com/Frontiers/SupplementaryVideo1.wmv)

**Video S2 | Examples of 20 different walking patterns.** AMOS II walks with different patterns from slow to fast speed with respect to *MI* = 0.0, 0.01, …, 0.19. (http://manoonpong.com/Frontiers/SupplementaryVideo2.wmv)

**Video S3 | Turning behavior.** AMOS II autonomously turns to avoid obstacles and escape from a corner and even a deadlock situation. It detects obstacles and a corner by using its ultrasonic sensors installed at front. (http://manoonpong.com/Frontiers/SupplementaryVideo3.wmv)

**Video S4 | Insect-like leg movements.** To clearly observe the insect-liked leg movements of AMOS II, we place it on a box and let it perform forward and backward walking. (http://manoonpong.com/Frontiers/SupplementaryVideo4.wmv)

**Video S5 | Leg elevation.** To clearly observe the leg elevation of AMOS II, we place it on a box and make the foot touch an obstacle during the swing phase. Due to mismatch between the expected foot contact signal, generated by the adaptive forward model, and the actual one, AMOS II can immediately elevate its leg (here right middle leg) to free the leg from the obstacle. In addition, we show that using an IR sensor at the leg also allows AMOS II to elevate its leg in order to avoid hitting the obstacle. The first part of this video corresponds to the result shown in Figures 7G–J of the manuscript. (http://manoonpong.com/Frontiers/SupplementaryVideo5.wmv)

**Video S6 | Searching for a foothold.** To clearly observe searching for a foothold of AMOS II, we place it on a box and change the ground level during the stance phase. Due to mismatch between the expected foot contact signal, generated by the adaptive forward model, and the actual one, AMOS II can immediately lowers its leg (here right middle leg) to search for a foothold. This video corresponds to the result shown in Figures 7C–F of the manuscript. (http://manoonpong.com/Frontiers/SupplementaryVideo6.wmv)

**Video S7 | Energy-efficient and adaptable locomotion on different terrains.** First test shows that AMOS II walks with a fast wave gait since it detects fine gravel (loose terrain) using its visual system. Afterward, it changes from the wave gait to a tetrapod gait since it detects gravel (rough terrain). Finally, it uses a fast tripod gait on the floor (hard terrain). Another test in an outdoor environment shows that AMOS II walks with a tetrapod gait since it detects gravel (rough terrain). Afterward, it changes from the tetrapod gait to a tripod gait since it detects grass (vegetated terrain). Note that during traversing the different terrains, AMOS II adapts its legs individually to the terrains. The first part of this video corresponds to the result shown in Figure 9 of the manuscript and the second part of this video corresponds to the result shown in Figure A4. (http://manoonpong.com/Frontiers/SupplementaryVideo7.wmv)

**Video S8 | Adaptable locomotion on terrain with small obstacles.** First test shows that AMOS II can free its right front leg after the leg hits an obstacle during the swing phase. Due to the difference of the ground level, AMOS II also adapts its legs by lowering them more downward to ensure ground contact during the stance phase. Other tests also show this kind of adaptable locomotion of AMOS II. The first part of this video corresponds to the result shown in Figure 10 of the manuscript. (http://manoonpong.com/Frontiers/SupplementaryVideo8.wmv)

**Video S9 | Climbing over a large obstacle in an outdoor environment.** AMOS II walks on rough terrain and then climbs over an 11 cm high obstacle. This video corresponds to the results shown in Figure 11 of the manuscript. (http://manoonpong.com/Frontiers/SupplementaryVideo9.wmv)

**Video S10 | Adaptable locomotion during leg damage.** While AMOS II is walking, we disconnect the motor power connector of the FTi-joint of its left middle leg to simulate leg damage. Local leg control allows AMOS II to adapt its legs to deal with the leg damage. As a result, it could still move forward without problem. This first part of the video corresponds to the results shown in Figure 12 of the manuscript. Another test is shown in the second part of the video. The third part of the video shows that AMOS II fails to cope with leg damage if local leg control is not activated. The last part of the video shows walking behaviors with low and high ground clearance during leg damage. (http://manoonpong.com/Frontiers/SupplementaryVideo10.wmv)

**Video S11 | Passive compliances of the joints and legs of AMOS II.** The joint compliance enables AMOS II to passively flex its legs to avoid damages when the environment changes. In addition, its leg compliance allows it to absorb external (ground reaction) forces. (http://manoonpong.com/Frontiers/SupplementaryVideo11.wmv)

**Video S12 | Walking and climbing like a cockroach.** AMOS II keeps its body very close to the ground during walking. While climbing, it uses its active backbone joint and leg adaptation. The video also compares its locomotion with cockroaches and other walking machines [four legs (Quadruped robot of the Stanford AI Lab, http://ai.stanford.edu), six legs (BILL-Ant-a robot of Case Biorobotics Lab, http://biorobots.cwru.edu/), eight legs (Scorpion robot of DFKI Bremen - Robotics Innovation Center, http://robotik.dfki-bremen.de/)]. Note that cockroach videos are referred to (Ritzmann et al., 2004; Abbott, 2007; Lewinger and Quinn, 2009) while the walking machine videos are referred to (Lee et al., 2006; Spenneberg and Kirchner, 2007; Lewinger and Quinn, 2009). (http://manoonpong.com/Frontiers/SupplementaryVideo12.wmv)

**Video S13 | Testing the adaptive neural locomotion controller on another six-legged walking machine.** We transfer the adaptive neural locomotion controller to another walking machine. We set *MI* of the CPG to 0.15; thereby, the controller generates a tripod gait with a walking frequency of approximately 0.8 Hz for the machine. As a result, the controller allows the machine to perform foothold searching when its leg loses ground contact, to adapt its locomotion to deal with irregular terrain or different ground levels, and to climb over a 7 cm high obstacle. For climbing, additional reactive active backbone joint control is also applied. (http://manoonpong.com/Frontiers/SupplementaryVideo13.wmv)

## Appendix

### The walking machine platform AMOS II (biomechanics)

The most important specification of AMOS II is presented in the main text of the manuscript. Therefore, we only provide here a clear picture of the active backbone joint (BJ) of AMOS II including its angle range (see Figure A1, left). Its minimum downward position (−45°) is comparable to the one observed in a cockroach (see Figure A1, right). Due to the mechanical design of the BJ, it allows the joint to also lean upwards to a maximum position of 45°. The leaning upward and downward motions are used for climbing over a large obstacle having a height up to 13 cm or 75% of the leg length of AMOS II.

### Complete neural circuit

Figure A2 shows the complete neural circuit of the adaptive locomotion controller. The controller generates versatile locomotion behavior of AMOS II by means of CPG-based control and local leg control (see main text for details). In total the controller has six neural modules where the modules I–IV belong to the CPG-based control and the modules V, VI belong to the local leg control.

**Module I (CPG with neuromodulation)**: *MI* = modulatory input; *C*_1,2_ = output neurons of the CPG. We use a hyperbolic tangent (tanh) transfer function for the CPG neurons.

**Module II (neural CPG postprocessing)**: *CP*_1,2_ = postprocessing neurons with a step function; *Int*_1,2_ = integrator units.

**Module III (neural motor control)**: *I*_1,…,4_ = neural control parameters for generating different walking directions and stopping motion; *H*_1,…,14_ = interneurons of the phase switching network (PSN); *H*_15,…,28_ = interneurons of the velocity regulating networks (VRNs). We use a tanh transfer function for the interneurons. Parameters are *A* = 1.7246, *B* = −2.48285, and *C* = −1.7246.

**Module IV (motor neurons)**: *M*_1,…,5_ = premotor neurons; *TR*_1_,*CR*_1_, *FR*_1_ = TC-, CTr- and FTi-motor neurons of the right front leg (R1); *TR*_2_, *CR*_2_, *FR*_2_ = right middle leg (R2); *TR*_3_, *CR*_3_, *FR*_3_ = right hind leg (R3); *TL*_1_, *CL*_1_, *FL*_1_ = left front leg (L1); *TL*_2_, *CL*_2_, *FL*_2_ = left middle leg (L2); *TL*_3_, *CL*_3_, *FL*_3_ = left hind leg (L3); *BJ* = a backbone motor neuron which is controlled by reactive BJ control [not shown here but see Goldschmidt et al. (2012)]; τ = ipsilateral lag (i.e., 16 time steps or ≈0.6 s); τ_*L*_ = the phase shift between both left and right sides (i.e., 48 time steps or ≈2 s). We use piecewise linear transfer functions for the premotor and motor neurons.

**Module V (adaptive neural forward models)**: *F*_1,…,6_ = adaptive hysteresis neurons for motor signal transformation; *W*_*I*_, *W*_*R*_, *B* = learning parameters; *P*_1,…,6_ = postprocessing neurons; Δ = an error between the expected foot contact (FC) signal and the actual one. We use a tanh transfer function for the hysteresis and postprocessing neurons.

**Module VI (searching and elevation control)**: *PD*_1,…,6_ = preprocessing neurons which provide only a positive error (+Δ); *ND*_1,…,6_ = preprocessing neurons which provide only a negative error (−Δ); *E*_1,…,6_ = *S*_1,…,6_ = recurrent neurons (i.e., accumulators). We use piecewise linear transfer functions for the preprocessing neurons and use a linear transfer function for the recurrent neurons.

Note that in all modules, all numbers are synaptic weights and the ones marked with subscript “B” refer to fixed bias terms.

Different exteroceptive and proprioceptive sensors are used here as inputs to the adaptive controller to generate stimulus induced behavior, energy-efficient gait, and adaptable locomotion. The sensors are: left and right ultrasonic sensors (US), six FC sensors (FC_1,…,6_), one USB camera (CM), six infrared reflex (IR) sensors (IR_1,…,6_), one current sensor (CS), and left and right light dependent sensors (LD). All raw sensory signals are preprocessed using neural preprocessing except the visual signal which is done by using an online feature-based terrain classification algorithm. This algorithm is briefly described in the main text of the manuscript.

We use a hysteresis neuron (*N*_1_) with a tanh transfer function for preprocessing the CS signal. The hysteresis principle (Pasemann, 1993) leads to a non-linear transition of two output states (low and high activations). Thus, hysteresis neuron can effectively filter sensory noise (Manoonpong et al., 2008b). The preprocessed CS signal which provides an energy level is used to inhibit all joint movements, thereby stopping robot motion, when the system has low power.

We use four neurons (*N*_2,…,5_) with a tanh transfer function to form the neural preprocessing network of the left and right LD signals and the left and right US signals. The network is developed based on a minimal recurrent controller (MRC) structure (Pasemann et al., 2003) which allows balancing positive (LD) and negative (US) tropisms. The network outputs (i.e., outputs of *N*_2,3_) provide orienting control signals which are transmitted to *I*_3,4_ of the neural motor control module. As a result, AMOS II can effectively perform an appropriate turning angle to avoid obstacles or corners as well as turn toward a light source.

We simply use neurons (*N*_6,…,17_) with a tanh transfer function for preprocessing the FC_1,…,6_ and IR_1,…,6_ signals. This is because the sensor signals contain small noise which can be eliminated by the non-linearity of the neuron. The preprocessed sensor signals are used for local leg control (described in the main text of the manuscript). All neural preprocessing parameters, e.g., synaptic strengths and bias terms (see Figure A2) were obtained by experiments [see Manoonpong et al. (2008a,b) for more details of the neural preprocessing parameters].

### Additional experimental results

Here we present three more experimental results that complement those shown in the main text of the manuscript.

Figure A3 shows 20 walking patterns with different speeds of AMOS II. These patterns are mainly controlled by the CPG-based controller. Setting the modulatory input *MI* of the CPG to 0.0, each leg steps in a wave on each side of the body with overlap. Increasing *MI*, stepping frequency increases and some legs steps in pairs (see dashed enclosures). This results in a variety of patterns (or gaits) including insect-like gaits and intermixed gaits. For example, one observes wave gaits with different frequencies (*MI* = 0.01–0.04), tetrapod gaits with different frequencies (*MI* = 0.05–0.06), caterpillar gaits with different frequencies (*MI* = 0.07–0.10), and tripod gaits with different frequencies (*MI* = 0.15–0.19). Legs are labeled from front to back as numbers 1–3 and the left and right sides are L and R, respectively. Note that increasing *MI* higher than 0.19, we found only two different gaits comparable to tripod gait (e.g., *MI* = 0.19) and caterpillar gait (e.g., *MI* = 0.10).

Figure A4 shows autonomous selection of energy-efficient gaits while traversing from gravel to grass in an outdoor environment. It can be seen that at the beginning AMOS II walked with a tetrapod gait (photos 1,2) since it detected gravel (rough terrain) using its visual system. Afterward, it changed from the tetrapod gait to a tripod gait (photo 3) since it detected grass (vegetated terrain). During traversing the different terrains, AMOS II adapted its legs individually to deal with a change of terrain. That is, it depressed its leg and extended its tibia to search for a foothold when losing a ground contact during the stance phase where this information is detected by a significant change of the positive accumulated error *o*_*S*_, see black line in Figure A4C. However, during the swing phase no leg elevation was observed since only minor perturbation occurred (i.e., no significant change of the negative accumulated error *o*_*E*_, see red line in Figure A4C). We encourage readers to see the video of this experiment at Video S7.

Figure A5 shows walking behaviors with high and low ground clearance when legs are damaged. In this test, AMOS II was driven by only the CPG-based control described in the section 2.3 of the manuscript. We let AMOS II walk with a slow wave gait (*MI* = 0.02) and then disconnected the motor power connectors of the CTr- and FTi-joints of the right (R3) and left (L3) hind legs and the left front leg (L1). The joints became inactive (i.e., uncontrollable). This is to simulate leg damage. It can be seen that AMOS II with high ground clearance had large body inclination (≈−18°, Figure A5A) leading to unstable locomotion and remaining legs need to carry more load. Thus, the motors need to produce high torque to carry the load resulting in high power consumption (Figure A5B). Furthermore the legs could not swing properly during swing phase (Figures A5C, D). In this case, the left middle leg (L2) always stayed on the ground; thereby, the robot turned to the left (Figure A5G and Video S10). In contrast, with low ground clearance the AMOS II fell down a little bit (Figure A5A) since its body was already close to the ground and remaining legs need not to carry more load leading to lower power consumption compared to the high ground clearance case (Figure A5B). The remanning legs (R1, R2, and L2) were able to swing during swing phase (Figures A5E, F). As a result, it could still move more straightforward compared to the high ground clearance case (Figure A5H and Video S10).
